# RINT1 deficiency disrupts lipid metabolism and underlies a complex hereditary spastic paraplegia

**DOI:** 10.1172/JCI162836

**Published:** 2023-07-17

**Authors:** Nathalie Launay, Montserrat Ruiz, Laura Planas-Serra, Edgard Verdura, Agustí Rodríguez-Palmero, Agatha Schlüter, Leire Goicoechea, Cristina Guilera, Josefina Casas, Felix Campelo, Emmanuelle Jouanguy, Jean-Laurent Casanova, Odile Boespflug-Tanguy, Maria Vazquez Cancela, Luis González Gutiérrez-Solana, Carlos Casasnovas, Estela Area-Gomez, Aurora Pujol

**Affiliations:** 1 Neurometabolic Diseases Laboratory, Institut d’Investigació Biomèdica de Bellvitge (IDIBELL), Hospital Duran i Reynals, L’Hospitalet de Llobregat, Barcelona, Spain.; 2 CIBERER, Centro de Investigación Biomédica en Red de Enfermedades Raras, ISCIII, Madrid, Spain.; 3 Pediatric Neurology unit, Department of Pediatrics, Hospital Universitari Germans Trias i Pujol, Universitat Autònoma de Barcelona, Spain.; 4 Research Unit on BioActive Molecules (RUBAM), Departament de Química Biomèdica, Institut de Química Avançada de Catalunya (IQAC-CSIC), Barcelona, Spain.; 5 CIBEREHD, Centro de Investigación Biomédica en Red de Enfermedades heoaticas y digestivas, ISCIII, Madrid, Spain; 6 ICFO-Institut de Ciencies Fotoniques, The Barcelona Institute of Science and Technology, Castelldefels, Spain.; 7 Laboratory of Human Genetics of Infectious Diseases, Necker Branch, Institut National de la Santé et de la Recherche Médicale, UMR 1163, Necker Hospital for Sick Children, Paris, France.; 8 University of Paris, Imagine Institute, Paris, France.; 9 St. Giles Laboratory of Human Genetics of Infectious Diseases, Rockefeller Branch, The Rockefeller University, New York, New York, USA.; 10 Pediatric Hematology-Immunology Unit, Necker Hospital for Sick Children, Paris, France.; 11 Howard Hughes Medical Institute, New York, New York, USA.; 12 CRMR Leukofrance Service de Neuropédiatrie, Hôpital Robert Debré AP-HP, Paris, France.; 13 UMR1141 Neurodiderot Université de Paris Cité, Paris, France.; 14 Pediatric Neurology Unit, Hospital Teresa Herrera, A Coruña, Spain.; 15 Consulta de Neurodegenerativas, Sección de Neurología Pediátrica, Hospital, Infantil Universitario Niño Jesús, Madrid, Spain.; 16 Neuromuscular Unit, Neurology Department, Hospital Universitari de Bellvitge, Universitat de Barcelona, L’Hospitalet de Llobregat, Barcelona, Spain.; 17 Department of Neurology, Columbia University, New York, New York, USA.; 18 Catalan Institution of Research and Advanced Studies (ICREA), Barcelona, Spain.

**Keywords:** Metabolism, Neuroscience, Mitochondria, Neurological disorders

## Abstract

The Rad50 interacting protein 1 (*Rint1*) is a key player in vesicular trafficking between the ER and Golgi apparatus. Biallelic variants in *RINT1* cause infantile-onset episodic acute liver failure (ALF). Here, we describe 3 individuals from 2 unrelated families with novel biallelic *RINT1* loss-of-function variants who presented with early onset spastic paraplegia, ataxia, optic nerve hypoplasia, and dysmorphic features, broadening the previously described phenotype. Our functional and lipidomic analyses provided evidence that pathogenic *RINT1* variants induce defective lipid–droplet biogenesis and profound lipid abnormalities in fibroblasts and plasma that impact both neutral lipid and phospholipid metabolism, including decreased triglycerides and diglycerides, phosphatidylcholine/phosphatidylserine ratios, and inhibited Lands cycle. Further, *RINT1* mutations induced intracellular ROS production and reduced ATP synthesis, affecting mitochondria with membrane depolarization, aberrant cristae ultrastructure, and increased fission. Altogether, our results highlighted the pivotal role of RINT1 in lipid metabolism and mitochondria function, with a profound effect in central nervous system development.

## Graphical abstract

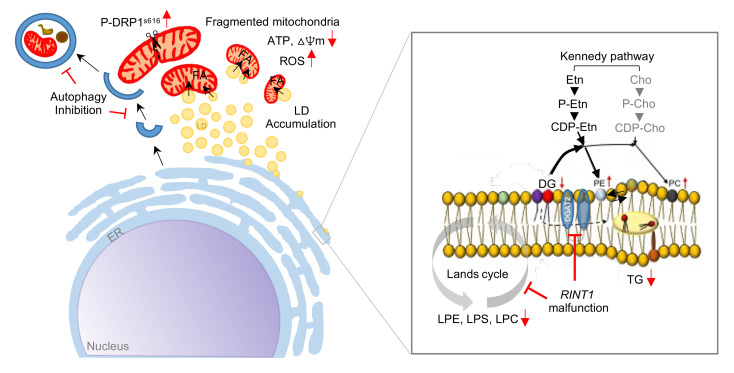

## Introduction

Lipid droplets (LDs) are cellular organelles that play a central role in the regulation of cellular metabolism, lipid homeostasis, and organelle dynamics (1, 2). Abnormalities in LDs have thus been associated with many pathologies, such as cardiovascular disease, cancer, obesity, fatty liver disease, and neurological disorders (3–7), including hereditary spastic paraplegias (HSP) (8–10). Human genetic studies have broadened our understanding of these disorders by uncovering a growing list of HSP genes involved in LD biogenesis and regulation (11–18). LDs originate from the ER and are composed of a hydrophobic neutral lipid core of triglycerides and cholesteryl esters enclosed by a phospholipid monolayer. While the exact molecular mechanisms underlying the biogenesis of LDs remain largely unknown, recent work has unveiled the multisubunit machinery of the LD-ER-associated tethering NBAS-RINT1-ZW10 (NRZ) complex, composed of Neuroblastoma amplified sequence (NBAS [MIM: 608025]), RAD50-interacting protein 1 (RINT1 [MIM: 610089]), and Zeste White 10 (ZW10 [MIM: 603954]) proteins, as a crucial element for LD growth and biogenesis (19, 20) and Golgi-ER trafficking (21, 22). Furthermore, the Rad50 Interacting Protein 1 (Rint1) multitasking protein is a pivotal regulator of the G2/M checkpoint in response to DNA damage and controls telomere elongation and centrosome duplication (23–25). RINT1 is essential for embryonic development in the mouse (26). Furthermore, its conditional inactivation in the CNS causes perinatal lethality and cortical defects due to genomic instability in neuronal precursors (27, 28). Specific inactivation of *RINT1* in retinal progenitors induced malformations of the optic nerve and blindness (27, 28), underscoring its critical role in neural development.

Recently, 3 patients harboring biallelic mutations in *RINT1* were reported, suffering from infantile liver failure syndrome-3 (ILFS3, OMIM no. 618641), which is an autosomal-recessive disorder characterized by recurrent episodes of acute liver failure (ALF) during intercurrent febrile illness. Other features in the clinical presentation included short stature and variable vertebral skeletal anomalies, while no defects in neurodevelopment and cognition were detected (29).

Here, we performed whole-exome sequencing (WES) in a cohort presenting with spastic paraplegia and identified pathogenic variants in *RINT1* in patients affected with early onset spasticity, ataxia, optic nerve hypoplasia, and neurodevelopmental delay that, to our knowledge, have not previously been reported. Our findings provide evidence for a fundamental contribution of RINT1 to early CNS development and highlight the emerging role of lipid disturbances and subsequent metabolic disruptions as drivers of severe multisystemic diseases.

## Results

### Clinical features and diagnosis of patients with a RINT1 mutation.

**Figure 1:**
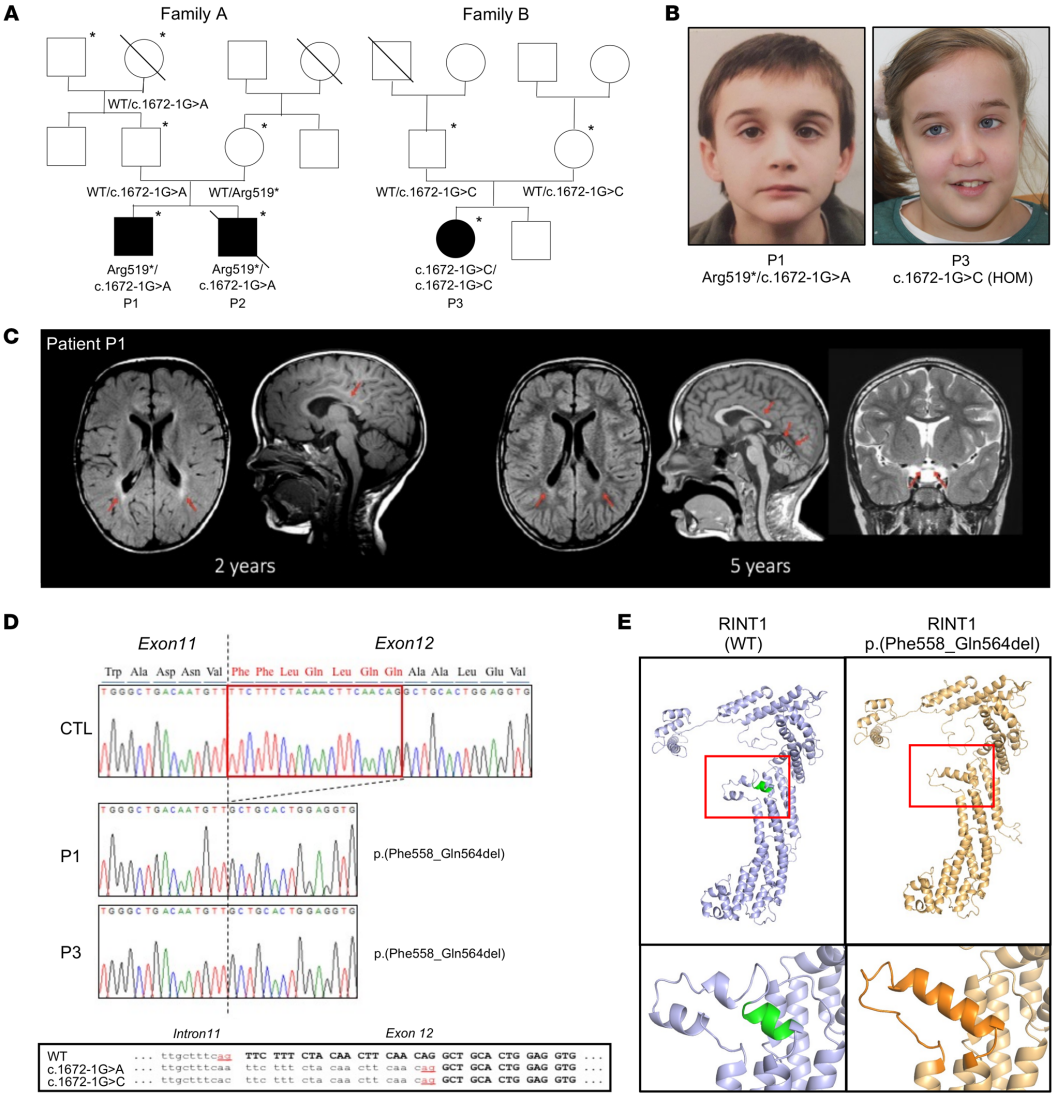
*RINT1* variant features. (**A**) Pedigrees of families A and B. Asterisks denote genotyped individuals. (**B**) Photograph illustrating dysmorphic features, notably low-set ears and strabismus, seen in patients P1 and P3. (**C**) Axial FLAIR and sagittal T1 MRI sequences of patient P1 showing posterior periventricular hyperintensities and thinning of the corpus callosum at 2 years (red arrows) and improvement of posterior periventricular white matter hyperintensity and signs of cerebellar atrophy compared with the previous study and optic chiasm atrophy (coronal T2 image) at 5 years (red arrows). (**D**) Sanger sequencing results for the RT–PCR products for a control individual and the patients revealing an in-frame deletion of 7 amino acids (p.Phe558_Gln564del) in patients P1 and P3. (**E**) 3D representations of the WT and mutated forms (p.Phe558_Gln564del) of RINT1. The structures were modelled with the Swiss-Prot server using the 3FHN model (Tripathi et al*.*, 2009) as a template (Tip20p, homologue in *S*. *cerevisiae*).

In the framework of a research project aiming to end the diagnostic odyssey in patients with suspected neurological diseases of genetic origin (URD-Cat, Undiagnosed Rare Diseases Consortium of Catalonia), we performed WES in a subset of patients from a cohort affected with spastic paraplegias and white-matter disorders (30). Here, we describe patients from 2 independent families that we examined (Figure 1A) in whom we found suspicious variants in RINT1.

Patients P1 and P3 showed remarkable phenotypic convergence, presenting an unreported progressive neurological phenotype characterized by complex spastic paraparesis associated with ataxia. A summary of the clinical features is given in Table 1, and detailed clinical case histories are provided in the Supplemental Materials. In patient P1, the pattern observed via MRI — mild cerebellar atrophy associated with subtle signal increases in T2 sequences — seemed to reflect a progressive neuronal disorder rather than leukodystrophy (31) (Figure 1C). Patients P1 and P3 also presented with nystagmus, poor visual acuity, and optic nerve hypoplasia. Patient P2 died at 14 months due to acute liver failure, probably before the development of a neurological phenotype.

The episodic liver dysfunction of patients P1 and P2 was very similar to that previously reported (29), and both siblings presented with viral infection-dependent episodes of ALF during infancy or childhood (at 14 months for P2 and 10 years of age for P1) that were associated with elevated AST and ALT levels. The postmortem examination of patient P2 revealed the presence of hepatic steatosis. Moreover, vacuolization of the proximal tubules of the kidneys, which may indicate renal dysfunction, was reported. The neurological involvement and facial dysmorphic traits, including anteverted nose, high-arched palate, wide forehead, and low-set ears, observed in patients P1 and P3 were, to our knowledge, not previously described in patients with a RINT1 mutation (29) (Figure 1B).

We applied WES for the probands of each family (P1, P2, and P3) and analyzed the data with our pipeline based on interactome networks (30). We identified novel biallelic variants in the *RINT1* gene in all 3 patients. Patients P1 and P2 harbored compound heterozygous variants (1 nonsense, 1 canonical splice-altering mutation) in *RINT1* (Chr7:105195558C>T, NM_021930.6:p.Arg519Ter; and Chr7:105204179G>A, NM_021930.6: c.1672-1G>A), and patient P3 harbored a homozygous canonical splice-altering variant (Chr7:105204179G>C, NM_021930.6: c.1672-1G>C) (Figure 1A). Of note, patients from both families inherited at least 1 canonical splice-altering variant in the same nucleotide, although with different changes: G>A and G>C. Variant frequencies were compatible with an autosomal recessive mode, and no homozygous-healthy individuals were present in the gnomAD database (Supplemental Table 1; supplemental material available online with this article; https://doi.org/10.1172/JCI162836DS1). No other variants of interest were detected by our pipeline. Sanger sequencing of RT–PCR products amplified from patient RNA revealed only 1 population of mutated transcripts, likely because in patients P1 and P2, the second allele was a nonsense mutation whose transcript was degraded by nonsense-mediated decay (Figure 1D). Strikingly, these transcripts contained an in-frame deletion of 7 amino acids (p.Phe558_Gln564del) that was observed in all patients. This deletion was the result of the destruction of the canonical acceptor site and the use of a secondary acceptor site 21 bp away inside exon 12. This may suggest the presence of a stable, hypomorphic allele encoding a transcript with residual function.

### Pathogenic variants alter NRZ complex subunit expression and localization in patient fibroblasts.

**Figure 2:**
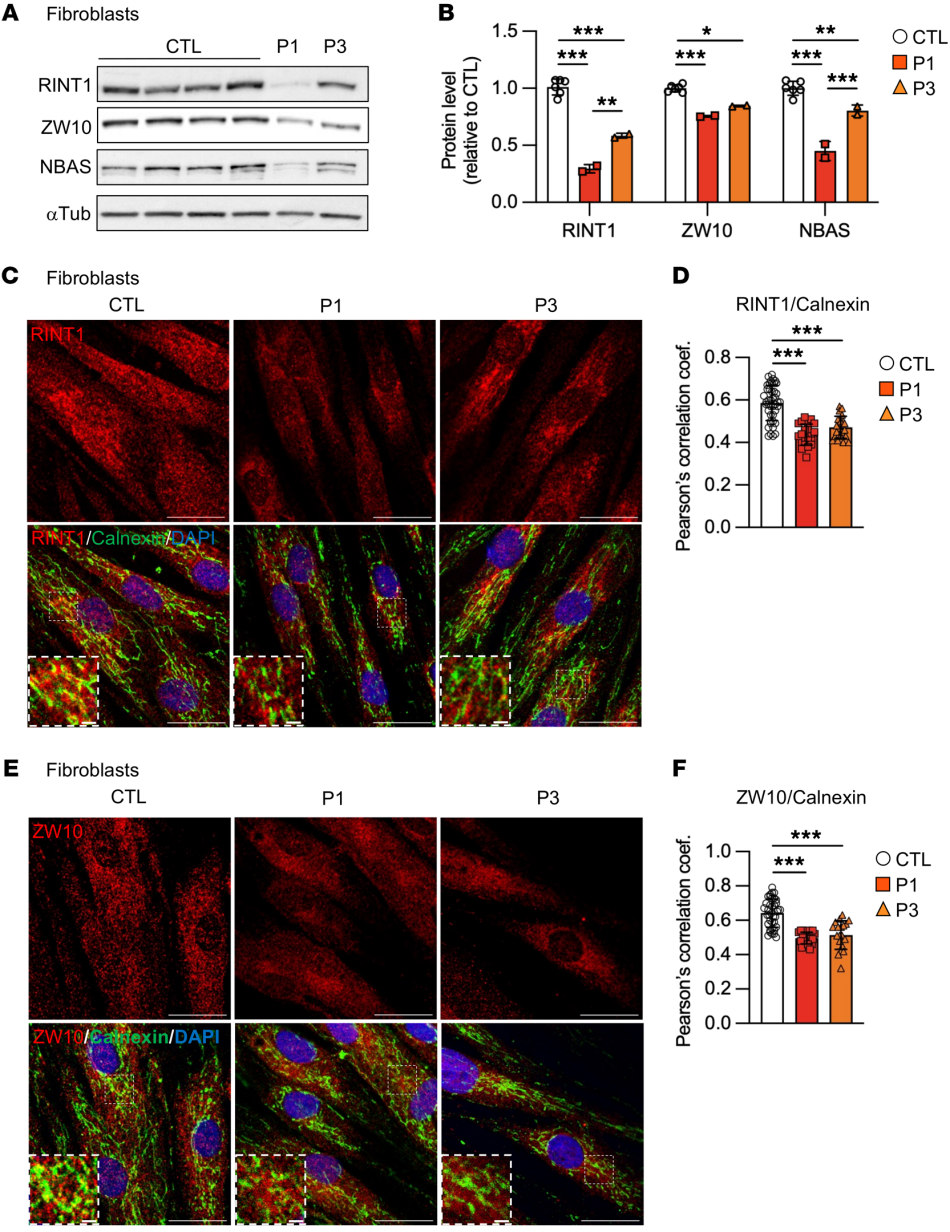
*RINT1* mutations alter NRZ complex. (**A**) Control (CTL) and patient (P1 and P3) fibroblasts were subjected to immunoblot analysis using the anti-RINT1, anti-ZW10, and anti-NBAS antibodies. The total amount of α-tubulin (α-tub) was used as a loading control. Blots run in parallel using identical samples are shown. (**B**) Quantification of RINT1, ZW10, and NBAS protein levels in patient fibroblasts (P1 and P3) relative to the controls (CTL, *n* = 6). (**C**–**F)** Representative confocal images of control (CTL) and patient (P1 and P3) fibroblasts stained with the anti-Calnexin and anti-RINT1 antibodies (**C**) or the anti-Calnexin and anti-ZW10 antibodies (**E**). Scale bars: 10 μm. A zoomed-in view is shown for each image with a scale bar of 2 μm. (**D** and **F**) Colocalization between RINT1 (**D**), ZW10 (**F**), and Calnexin is expressed as Pearson’s coefficient measured for individual cells. *n* > 20 cells for each genotype. Patient (P1 and P3) and control (CTL, *n* = 3) fibroblasts. All data are shown as the mean ± SD. Results were obtained from 2 independent experiments. *P < 0.05, **P < 0.01, ***P < 0.001. All data analysis were performed using 1-way ANOVA followed by Tukey’s test for multiple comparisons.

In agreement with the previously published structure of the RINT1/Tip20 protein from *S*. *cerevisiae* (32), we used structural analysis in silico with the Swiss-Prot server to assess the effects of *RINT1* mutations (p.Phe558_Gln564del) on protein structure (33). We predicted that these *RINT1* mutations created an helix bundle in the core structure that may be detrimental for protein stability (Figure 1E). In agreement, Western blot analysis in patient fibroblasts showed that RINT1 levels were significantly decreased in patients 1 and 3 (Figure 2, B and C). We next sought to determine whether RINT1 variants affected the protein levels of the other NRZ complex components, ZW10 and NBAS. Indeed, Western blot analysis showed that ZW10 and NBAS levels were also significantly decreased in fibroblasts from both patients (Figure 2, A and B), with a correlation between RINT1 abundance and NBAS levels (Figure 2, A and B).

Given that RINT1 has been shown to interact with syntaxin 18, a soluble N-ethylmaleimide-sensitive factor attachment protein receptor (SNARE) protein localized to the ER (25, 26, 34), we next tested the effect of *RINT1* variants on its colocalization with the ER marker Calnexin. We confirmed the localization of RINT1 to the ER in control fibroblasts, as described previously in HeLa and U2OS cells (25, 26, 34). In agreement with our Western blot data, the mutated RINT1 showed decreased fluorescence intensity and reduced colocalization with ER markers in patient’s cells (Figure 2, C and D). Moreover, we detected a reduction of ZW10 localization in the ER and its redistribution to perinuclear areas compared with controls (Figure 2, E and F).

Altogether, these findings suggest that pathogenic variants in *RINT1* may destabilize the NRZ complex by altering the expression and localization of its components.

### RINT1 mutations lead to autophagy inhibition and Golgi morphology abnormalities.

**Figure 3:**
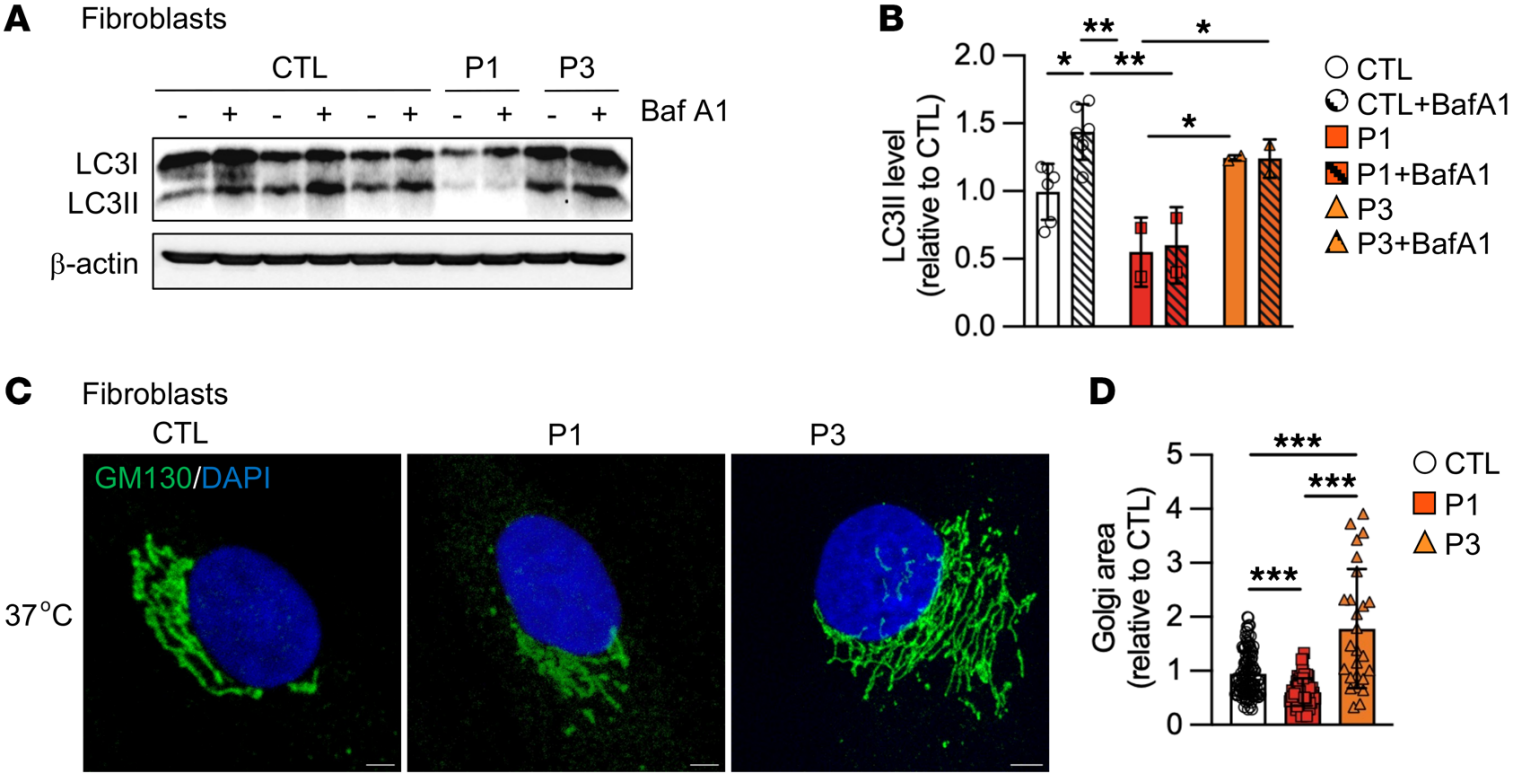
*RINT1* mutations impair Autophagic Flux and lead to abnormal Golgi morphology. (**A**) Representative Western blot showing LC3-II levels from control (CTL) and patient (P1 and P3) fibroblasts without or with Bafilomycin A1 (Baf A1). The total amount of β-actin was used as a loading control. (**B**) Quantification of LC3-II levels in patient fibroblasts (P1 and P3) relative to the controls (CTL, *n* = 6). (**C**) Representative images of Golgi apparatus of control (CTL) and patient (P1 and P3) fibroblasts incubated at 37°C. Scale bars: 5 μm. (**D**) Quantification of Golgi area in patient fibroblasts (P1 and P3) relative to control cells (CTL, *n* = 3). *n* > 50 cells for each genotype. All data are shown as the mean ± SD. Results were obtained from 2 independent experiments. *P < 0.05, **P < 0.01, ***P < 0.001. Analysis of data in **B** was performed using 2-way ANOVA followed by Tukey’s test for multiple comparisons. Data in **D** were analyzed using 1-way ANOVA followed by Tukey’s test for multiple comparisons.

The initial report of RINT1-linked ILFS3 showed an impaired autophagic flux and Golgi apparatus abnormalities (29). We thus investigated whether the identified variants led to similar effects. First, autophagic flux was estimated by the change in Microtubule-associated protein 1A/1B-light chain 3-II (LC3-II) amount under the treatment with bafilomycin A1, an inhibitor of the lysosomal V-ATPase. As expected, control cells showed a significant increase in the levels of LC3-II in the presence of the autophagy inhibitor. In contrast, no change in LC3-II levels was detected upon bafilomycin A1 in patient’s fibroblasts, implying that the autophagic process in RINT1-mutated cells was impaired (Figure 3, A and B). Importantly, the patient P1 presented with very low levels of LC3-II at baseline, suggesting an important alteration of autophagosome biogenesis (Figure 3, A and B).

To determine whether Golgi structural features were altered by the identified *RINT1* mutations, we examined the morphology of Golgi in our cell models by confocal microscopy. As measured by volume projections of cells stained with antibodies against the *cis*-Golgi marker GM130, patient’s P1 cells presented with a decrease in Golgi volume compared with control cells. In contrast, patient P3 fibroblasts exhibited an expanded Golgi network, similar to previous descriptions in 2 patients by Cousin et al. (29) (Figure 3, C and D). No significant change in Golgi morphology was observed at temperature in the febrile range (40°C) versus 37°C (Supplemental Figure 3, A and B).

### RINT1 variants result in accumulation of smaller lipid droplets in patient’s fibroblasts.

**Figure 4:**
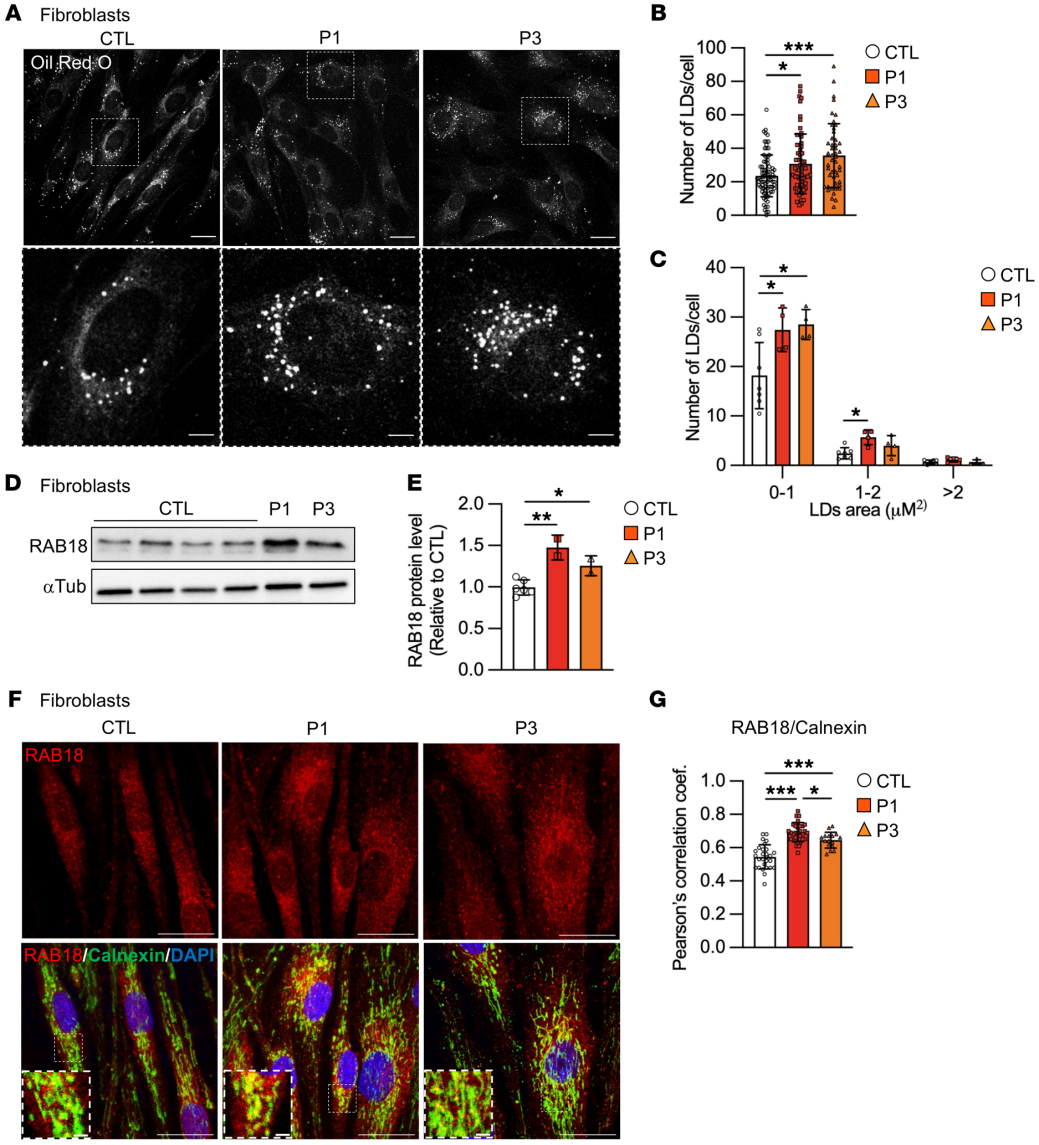
*RINT1* mutations alter LD size and number. (**A**) Representative images of LDs in control (CTL) and patient (P1 and P3) fibroblasts at the basal level. LDs were stained with Oil red O. Scale bars: 20 μm. A zoomed-in view is shown for each image with a scale bar of 2 μm. (**B** and **C**) Quantification of the number (**B**) and average area (**C**) of LDs/cell observed in panel **A**. *n* > 50 cells for each genotype. Patient (P1 and P3) and control (CTL, *n* = 3) fibroblasts. (**D**) Control (CTL) and patient (P1 and P3) fibroblasts were subjected to immunoblot analysis using the anti-RAB18 antibody. The total amount of α-tubulin (α-tub) was used as a loading control. (**E**) Quantification of RAB18 protein level in patient (P1 and P3) fibroblasts relative to the controls (CTL, *n* = 6). (**F**) Representative confocal images of control (CTL) and patient (P1 and P3) fibroblasts labeled with the anti-Calnexin and anti**-**RAB18 antibodies. Scale bars: 10 μm. A zoomed-in view is shown for each image with a scale bar of 2 μm. (**G**) Colocalization between RAB18 and Calnexin is expressed as Pearson’s coefficient measured for individual cells. *n* > 20 cells for each genotype. Patient (P1 and P3) and control (CTL, *n* = 3) fibroblasts. All data are shown as the mean ± SD. Results were obtained from 2 independent experiments. *P < 0.05, **P < 0.01, ***P < 0.001. The data in **B, E**, and **G** were analyzed by 1-way ANOVA followed by Tukey’s test for multiple comparisons. The data in **C** were analyzed by 2-way ANOVA followed by Tukey’s test for multiple comparisons.

Depending on cellular needs, LDs can grow, shrink, and/or interact with various organelles (35). Depletion of NBAS or ZW10 in 3T3-L1 preadipocytes led to the accumulation of large LDs (20). However, in COS7 cells with *ZW10* knockdown, LDs appeared smaller and more abundant compared with controls (36). This suggests that the inactivation of the NRZ complex affects LDs biogenesis, although the cellular context governs the final phenotype. We thus set out to investigate the potential effect of *RINT1* variants on LD biogenesis by measuring LD number and size in patient fibroblasts. Oil Red O staining visualized by confocal microscopy showed that the number of LDs was significantly increased in RINT1-mutated cells (Figure 4, A and B), although their size was significantly smaller (Figure 4, A and C), suggesting a defect in LD biogenesis and growth.

For an improved understanding of the mechanisms involved in these LD alterations, we determined the levels and localization of the small Ras-family GTPase RAB18, previously reported to drive the association of LDs with ER membranes. The Rab18-NRZ-SNARE interaction and complex formation results in the tethering of the ER to LDs, promoting LD growth and lipid storage (19, 20). Of note, we found higher RAB18 protein levels in patient’s cells compared with controls (Figure 4, D and E). Moreover, mutant fibroblasts showed an increased colocalization between RAB18 and Calnexin (Figure 4, F and G), suggesting that dismantling the NRZ complex may promote stronger RAB18-ER interactions.

### RINT1 malfunction disrupts lipid homeostasis.

The synthesis and storage of lipids in the ER is essential in the regulation of LD growth, as well as to the overall maintenance of lipid homeostasis. In the ER, the enzymes acyl-CoA diacylglycerol acyltransferase 1 and 2 (DGAT1 and DGAT2) catalyze the final step in the synthesis of triacylglycerols (TGs) (37). Together with cholesteryl esters (CEs), TGs constitute the core of the LD, which is enclosed by a phospholipid monolayer (38).

To determine the impact of RINT1 malfunction in systemic lipid homeostasis, we next carried out a lipidomic analysis by liquid chromatography-high resolution mass spectrometry (LC-HRMS) of fibroblasts and plasma from patients P1 and P3.

**Figure 5:**
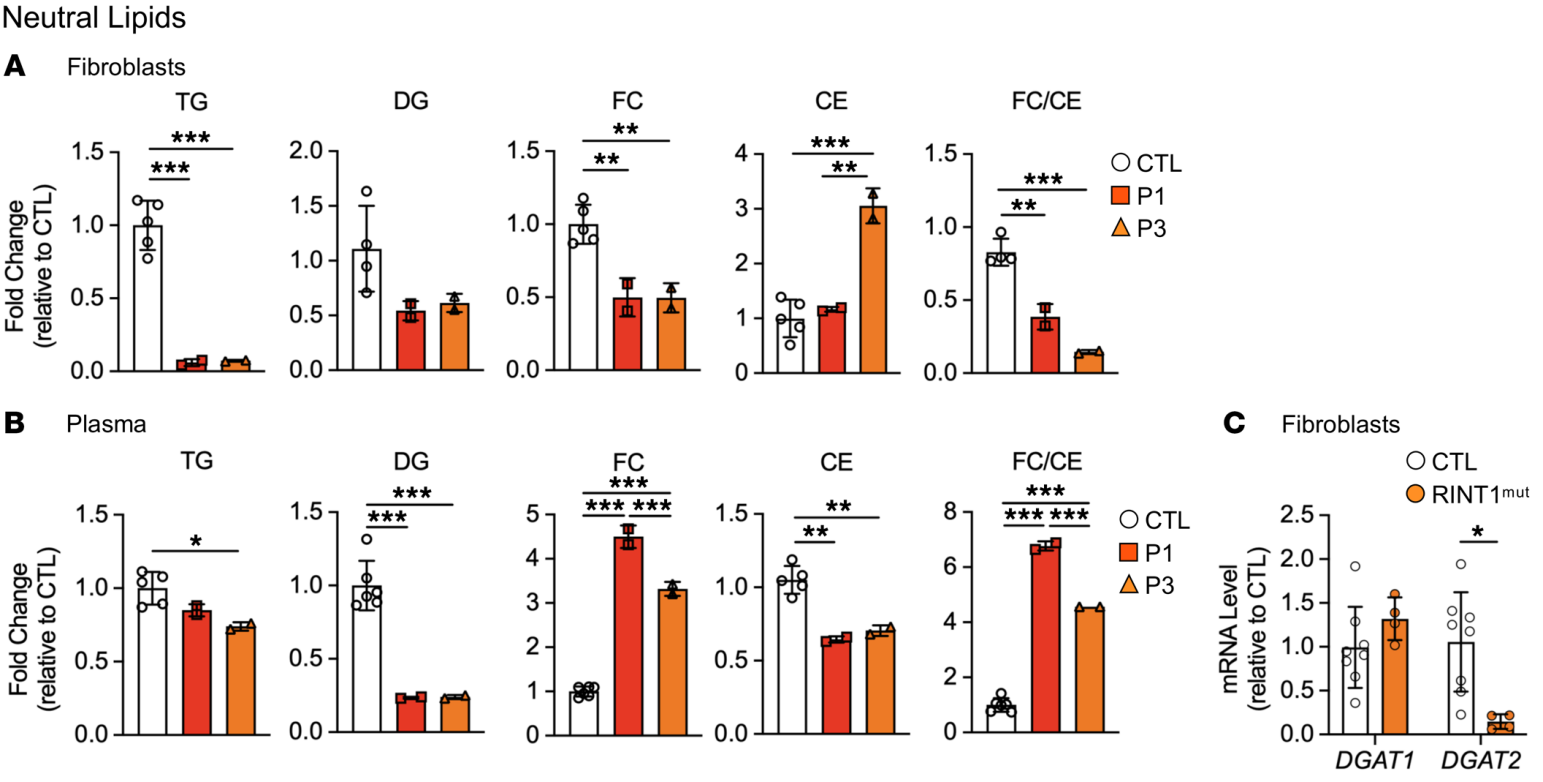
Impaired TG synthesis in fibroblasts and plasma from patients with *RINT1* mutations. (**A** and **B**) Lipidomic analysis of total neutral lipids in fibroblasts (**A**) and plasma (**B**) from patients P1 and P3 relative to control individuals (CTL, *n* = 5-6) (triacylglycerols (TG); diacylglycerols (DG); free cholesterol (FC); cholesterol esters (CE) and FC/CE ratio). (**C**) mRNA levels of *DGAT1* and *DGAT2* in patient fibroblasts (RINT1^mut^, *n* = 2) relative to the control fibroblasts (CTL, *n* = 4). All data are shown as the mean ± SD. Results were obtained from 2 independent experiments. *P < 0.05, **P < 0.01, ***P < 0.001. Analysis of data in **A** and **B** were performed using 1-way ANOVA followed by Tukey’s test for multiple comparisons. Data in **C** were analyzed using unpaired 2-tailed *t* test.

Intriguingly, TG levels were decreased in mutant fibroblasts and plasma samples compared with control values (Figure 5, A and B and Supplemental Figure 1, A and B). In addition, the DG levels were also lower in fibroblasts and plasma samples from these patients compared with controls (Figure 5, A and B and Supplemental Figure 1, C and D). Free cholesterol (FC) and CE composition analysis of control and patient fibroblasts revealed a significant decrease in the FC/CE ratio in patient cells, suggesting a higher cholesterol esterification (Figure 5A). Conversely, we found increased FC levels and FC/CE ratios in these plasma samples from patients with a RINT1 mutation (Figure 5B). Collectively, these results suggest a potential alteration in cholesterol turnover, as described in other cases of disturbed TG metabolism (39).

We next evaluated the effect of *RINT1* variants on TG synthesis enzymes and examined the expression of *DGAT1* and *DGAT2*. While the *DGAT1* levels were not significantly different, we found a drastic reduction in *DGAT2* mRNA expression levels in patient cells compared with control mRNA levels (Figure 5C).

**Figure 6:**
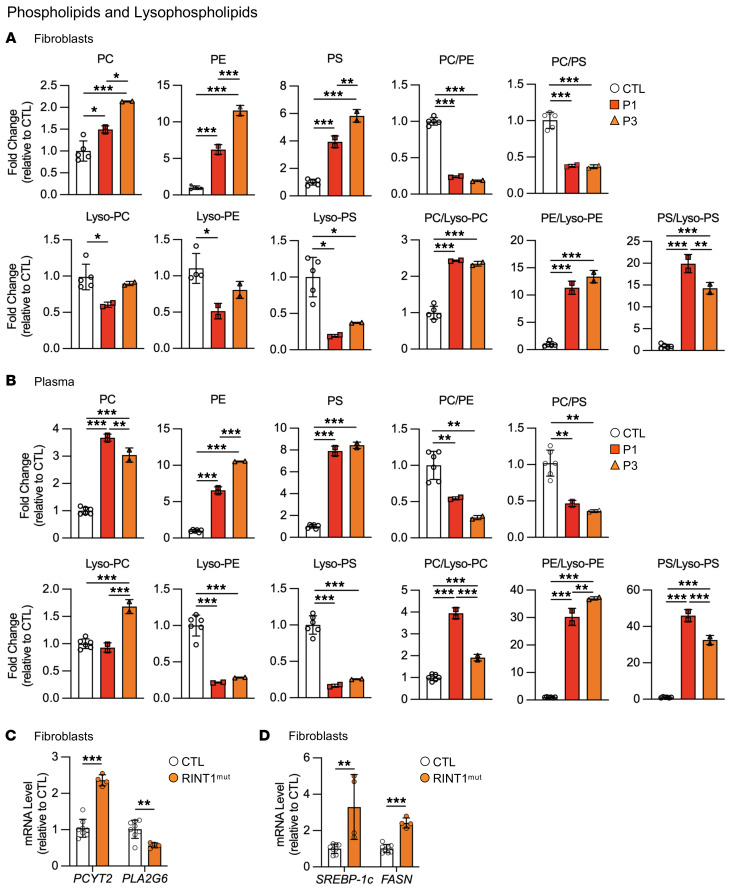
Oversynthesis of phospholipids in fibroblasts and plasma from patients with *RINT1* mutations. (**A** and **B**) Lipidomic analysis of total phospholipids and lysophospholipids in fibroblasts (**A**) and plasma (**B**) from patients (P1 and P3) relative to control (CTL, *n* = 5–6) individuals (phosphatidylcholine (PC); phosphatidylethanolamine (PE); phosphatidylserine (PS); PC/PE ratio; PC/PS ratio; lysophosphatidylcholine (Lyso-PC); lysophosphatidylethanolamine (Lyso-PE); lysophosphatidylserine (Lyso-PS); PC/Lyso-PC ratio; PE/Lyso-PE ratio; and PS/Lyso-PS ratio). (**C**) Increased *PCYT2* and decreased *PLA_2_G6* expression in patient fibroblasts (RINT1^mut^, *n* = 2) relative to control (CTL, *n* = 4) cells. (**D**) Patient fibroblasts (RINT1^mut^, *n* = 2) exhibited higher expression levels of *SREBP-1c* and *FASn* than control (CTL, *n* = 4) cells. All data are shown as the mean ± SD. Results were obtained from 2 independent experiments. *P < 0.05, **P < 0.01, ***P < 0.001. Analysis of data in **A** and **B** were performed using 1-way ANOVA followed by Tukey’s test for multiple comparisons. Data in **C** and **D** were analyzed using unpaired 2-tailed *t* test.

Depending on their needs, cells can use DGs as substrates for either the synthesis of TGs or phospholipids (40). We thus quantified the phosphatidylcholine (PC), phosphatidylethanolamine (PE), and phosphatidylserine (PS) levels in cells and plasma from patients. Our results showed that the overall phospholipid content was significantly increased in fibroblasts and plasma from patients with a RINT1 mutation compared with control levels (Figure 6, A and B and Supplemental Figure 2, A–F). Specifically, PC levels were approximately 1.8- and approximately 3.3-fold higher in fibroblasts and plasma from patients, respectively. Elevations in PE content reached approximately 6- to 10-fold in RINT1 patient cells and plasma. Similarly, PS levels were approximately 4- to 8-fold higher in RINT1 patient fibroblasts and plasma. As a result of these alterations, the PC/PE and PC/PS ratios drastically decreased in patient fibroblasts and plasma (Figure 6, A and B). These observations suggest that defects in RINT1 may result in the inhibition of DGAT2 and subsequent redirection of DG molecules toward the synthesis of phospholipids, particularly toward the formation of PE via the CDP-ethanolamine pathway.

Consistent with this increase in phospholipid levels, the concentration of lysophospholipid derivatives, mainly lysophosphatidylethanolamine (Lyso-PE) and lysophosphatidylserine (Lyso-PS), were decreased in both fibroblasts and plasma samples from patients with a RINT1 mutation (Figure 6, A and B and Supplemental Figure 2, G–L). As a result, the PC/Lyso-PC, PE/Lyso-PE and PS/Lyso-PS ratios were increased in the fibroblasts and plasma from both patients (in fibroblasts, PC/Lyso-PC: 2.4 ± 0.01- and 2.3 ± 0.06-fold; PE/Lyso-PE: 14.03 ± 1.4- and 16.54 ± 1.4-fold; and PS/Lyso-PS: 19.9 ± 2.1- and 14.2 ± 1.3-fold) (in plasma, PC/Lyso-PC: 3.94 ± 1.9 - and 1.91 ± 0.1-fold; PE/Lyso-PE: 30.2 ± 3.1- and 36.9 ± 0.5-fold; and PS/Lyso-PS: 45.9 ± 3.3- and 32.5±2.5-fold) (Figure 6, A and B).

This increase in phospholipid levels may either be the consequence of the activation of the de novo and salvage phospholipid synthesis pathways (Kennedy pathway and base-exchange reactions at mitochondria-associated ER membrane [MAM] domains, respectively) or the reacylation pathway (the Lands cycle). In the remodeling pathway, the cycle of phospholipid deacylation and reacylation modifies the fatty acid (FA) composition of phospholipids to adapt cell membranes to changes in their environment (41, 42). To test for these 2 possibilities, we analyzed the mRNA expression levels of key regulatory enzymes from these 2 pathways: *PCYT2* and *PLA2G6*. The *PCYT2* gene encodes the CTP:phosphoethanolamine cytidylyltransferase (ET), an ubiquitously expressed rate-limiting enzyme for PE synthesis via CDP-ethanolamine (Kennedy pathway) (43). The *PLA2G6* gene encodes the calcium-independent phospholipase A2 (iPLA_2_β), which belongs to the PLA_2_ superfamily that hydrolyses the sn-2 acyl chain of glycerophospholipids to release free FAs and lysophospholipids via the Lands cycle (44, 45). Interestingly, we found a marked increase in *PCYT2* expression in fibroblasts from patients with a RINT1 mutation, whereas that of *PLA2G6* was strongly downregulated (Figure 6C). All together, our data suggest that RINT1 defects result in a shift toward the use of DGs to synthesize phospholipids and inhibit the Lands cycle.

In support of this idea, the expression of sterol regulatory element binding protein-1 (SREBP-1c), a transcription factor that induces phospholipid biosynthesis by both direct and indirect mechanisms (46), and its target gene *FASN,* were significantly increased in patient fibroblasts (Figure 6D). These findings indicate a global dysregulation of lipid homeostasis in patients with a RINT1 mutation.

### RINT1 loss induces mitochondrial dynamics and bioenergetic defects.

Upon nutrient stress, such as fasting or glucose starvation, cells can shift from glycolysis to mitochondrial fatty acidβ-oxidation for ATP production. This change in carbon sources requires the transfer of FAs into the mitochondria, and it requires the remodeling of mitochondria into highly connected networks by modulating mitochondrial fission/fusion dynamics (47–50). Thus, mitochondrial fusion has been shown to be associated with FA oxidation derived from LDs in mouse fibroblasts (51, 52).

**Figure 7:**
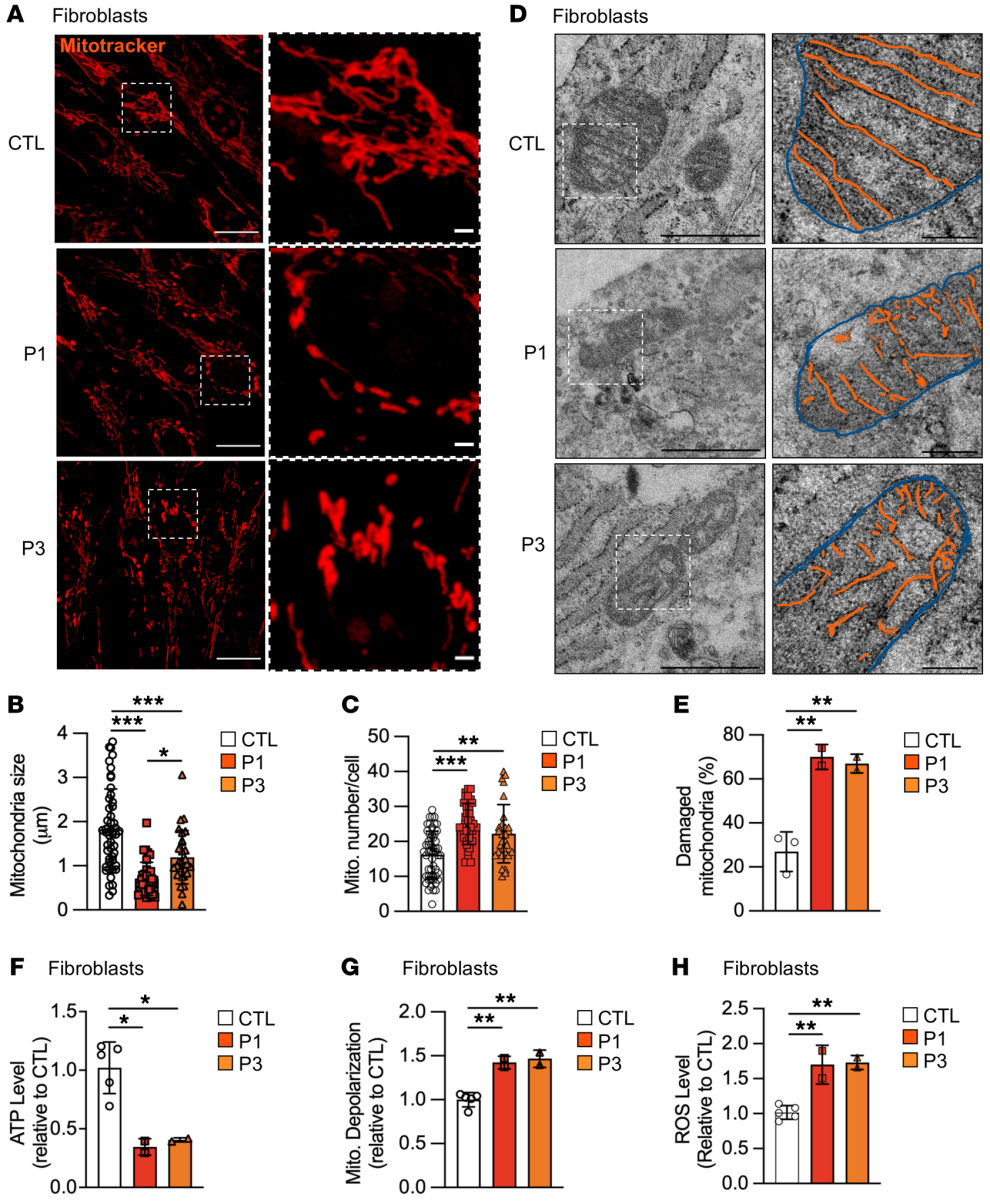
Pathogenic *RINT1* variants lead to mitochondrial abnormalities. (**A**) Representative images of mitochondrial network stained with MitoTracker Orange from control (CTL) and patient (P1 and P3) fibroblasts. Scale bar: 20 μm. A zoomed-in view is shown for each image; scale bar: 2 μm. (**B**) Quantification of the average mitochondrial size in control (CTL, *n* = 3) and patient (P1 and P3) fibroblasts. *n* > 20 cells for each genotype. (**C**) Quantification of mitochondrial number per cell in control (CTL, *n* = 3) and patient (P1 and P3) fibroblasts. *n* > 20 cells for each genotype. (**D**) Representative electron microscopy images displaying mitochondrial ultrastructure in control (CTL) and patient (P1 and P3) fibroblasts. Scale bar: 1 μm. A zoomed-in view is shown for each image; scale bar: 0.2 μm. Inter membrane space: blue; cristae: orange. (**E**) Percentage of damaged mitochondria in control (CTL, *n* = 3) and patient (P1 and P3) fibroblasts. *n* > 40 cells for each genotype. (**F**–**H**) ATP content (**F**) and depolarized mitochondrial (**G**) levels in patient (P1 and P3) fibroblasts compared with control (CTL, *n* = 5) fibroblasts. (**H**) Quantification of the intracellular ROS using the H2DCFDA probe in patient (P1 and P3) fibroblasts compared with control (CTL, *n* = 5) fibroblasts**.** All data are shown as the mean ± SD. Results were obtained from 2 independent experiments. *P < 0.05, **P < 0.01, ***P < 0.001. Analysis of data were performed using 1-way ANOVA followed by Tukey’s test for multiple comparisons.

To gain a better understanding of the bioenergetic homeostasis in RINT1 cells, we next analyzed the morphology of the mitochondrial network in cells from patients using MitoTracker Orange and an anti-TOMM20 antibody. As depicted in Figure 7A, mitochondria in control fibroblasts were elongated and appeared interconnected. In contrast, mitochondria in patient fibroblasts appeared individually distinct, smaller, and more rounded (called fragmented mitochondria), suggesting an impairment in mitochondria dynamics (Figure 7, A–C). TOMM20 staining showed a similar fragmentation pattern as MitoTracker Orange (Supplemental Figure 3C). No changes in the levels of mitochondrial markers VDAC and Complex III and IV subunits, used as indicators of mitochondrial content, were observed in mutant cells (Supplemental Figure 3, D and E). Moreover, transmission electron microscopy (TEM) analysis showed regular distribution of thin- and parallel-oriented cristae in mitochondria in control cells, whereas a high percentage of disorganized cristae was observed in patient fibroblasts (Figure 7, D and E). Collectively, these results indicate that *RINT1* mutations lead to impaired mitochondrial dynamics and aberrant cristae structure.

We investigated whether ATP levels and the mitochondrial membrane potential (ΔΨm), which are both indicators of preserved mitochondrial health (53, 54) and are severely impacted by altered dynamics, were intact. We observed that RINT1 fibroblasts exhibited both lowered levels of ATP (Figure 7F), enhanced mitochondrial depolarization (Figure 7G), and increased mitochondrial ROS levels (Figure 7H).

**Figure 8:**
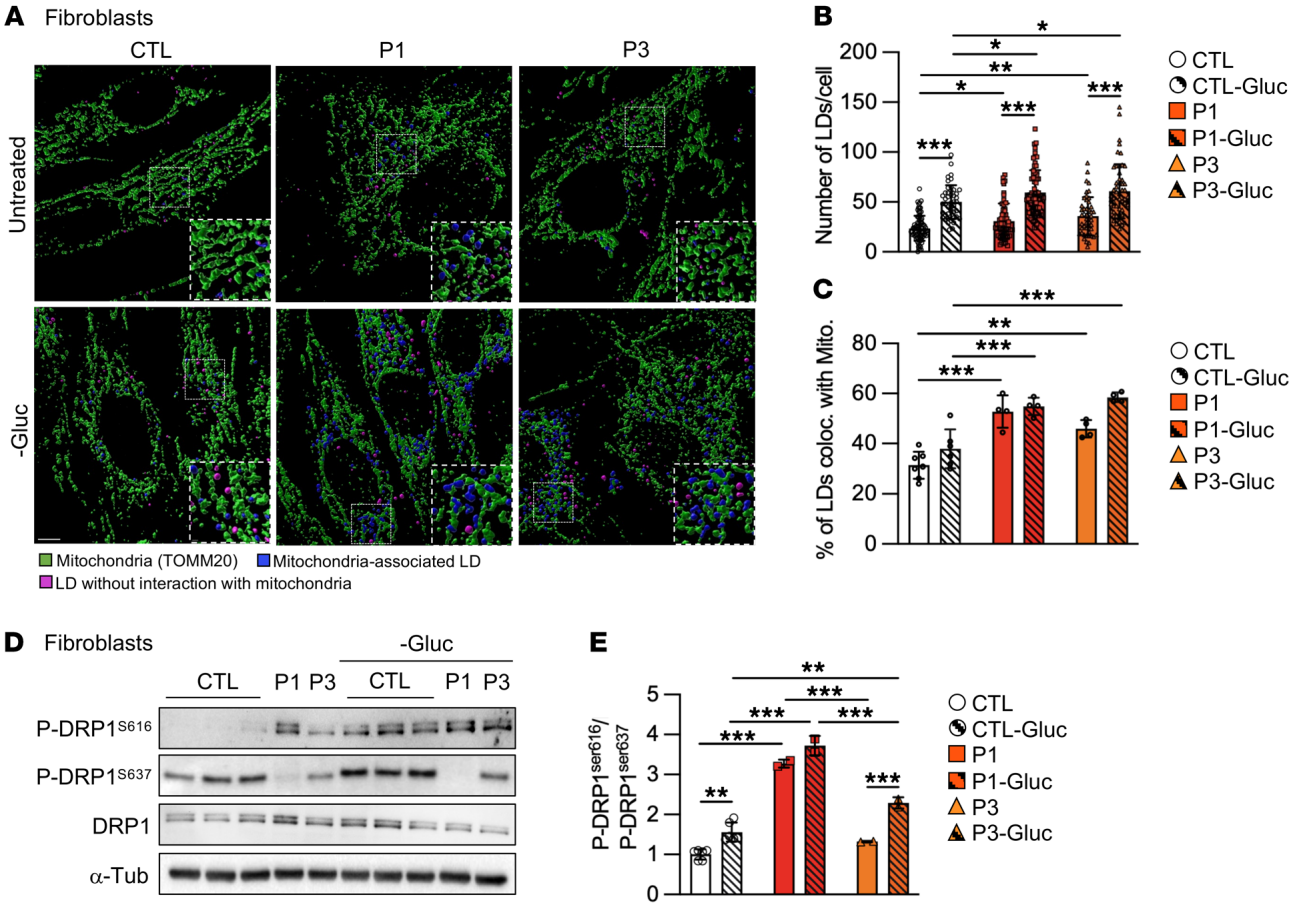
Glucose deprivation promotes LD accumulation and increases mitochondrial fragmentation. (**A**) 3D rendering of a confocal image stack of control (CTL) and patient (P1 and P3) fibroblasts incubated with glucose (untreated) or without glucose (−Gluc) for 16 hours. Cells were labelled with an anti-TOMM20 antibody (mitochondria) and oil red O (LDs), and Imaris analysis was applied to detect LD-mitochondria surface contacts. Scale bar: 5 μm. A zoomed-in view is shown for each image; scale bar: 0.7 μm. (**B**) Quantification of the number of LDs per cell in the presence and absence of glucose. *n* > 50 cells for each genotype and condition. Patient (P1 and P3) and control (CTL, *n* = 3) fibroblasts. (**C**) Quantification of the percentage of LDs in contact with mitochondria per cell in control (CTL) and patient (P1 and P3) fibroblasts in the presence or absence of glucose. *n* > 50 cells for each genotype and condition. CTL=3. (**D**) Representative immunoblots of P-DRP1^s616^, P-DRP1^s637^, and DRP1 protein levels in control (CTL) and patient (P1 and P3) fibroblasts incubated with or without glucose (–Gluc). The total amount of α-tubulin (α-tub) was used as a loading control. (CTL *n*=6). Blots run in parallel using identical samples are shown. (**E)** Quantification of the P-DRP1^S616^/ P-DRP1^S637^ ratio relative to the control cells. All data are shown as the mean ± SD. Results were obtained from 2 independent experiments. *P < 0.05, **P < 0.01, ***P < 0.001. The data were analyzed by 2-way ANOVA followed by Tukey’s test for multiple comparisons.

We next investigated the effect of nutrient stress on LD dynamics by incubating fibroblasts in the presence or absence of glucose. Starvation induced a marked increase in LDs in all cells, although this increase was significantly higher in fibroblasts from patients with a RINT1 mutation (Figure 8, A and B). Since LDs provide FAs to mitochondria upon glucose starvation, we next quantified the LD number and surface area in close proximity with mitochondria by analyzing 3D confocal images with Imaris software. In the presence or absence of glucose, mutant cells displayed a higher percentage of LDs closely positioned near mitochondria compared with controls, suggesting that FA transfer that occurs at mitochondria-LD contact sites is increased in RINT1-deficient cells (Figure 8, A and C).

In agreement with Rambold, et al. (52), we hypothesized that an imbalance in mitochondrial dynamics may lead to reduced FA oxidation, resulting in the accumulation of LDs in mutant cells. Mitochondrial fission is mainly driven by the GTPase dynamin-related protein 1 (DRP1), which promotes the generation of smaller-sized daughter mitochondria that are essential for mitochondrial transport, mitophagy, and apoptosis (55, 56). DRP1 recruitment to mitochondria is tightly regulated by several posttranslational modifications. In particular, phosphorylation at serine 616 (DRP1^S616^) induces the GTPase activity of DRP1 and promotes mitochondrial fragmentation (57), while phosphorylation of DRP1 at serine 637 (DRP1^S637^) inhibits its GTPase activity (58, 59). In response to nutrient starvation conditions, formation of an inter-connected mitochondrial network has been proposed to prevent excessive loss of mitochondria by creating a physical barrier to autophagy (60). Thus, under glucose starvation, Protein kinase A (PKA) phosphorylates Drp1 at serine 637 to counteract the mitochondrial fission (60, 61), preserving the mitochondrial network integrity and cell survival. Therefore, we investigated the expression pattern of mitochondrial fission proteins (i.e., DRP1, P-DRP1^S616^, and P-DRP1^S637^) in control and patient fibroblasts in the presence and absence of glucose. Consistent with an increase in mitochondrial fragmentation, we found that the P-DRP1^S616^/P-DRP1^S637^ ratio was significantly increased in patient fibroblasts at baseline (Figure 8, D and E). In addition, we found that, while glucose deprivation resulted in the activation of DRP1-mediated fission in control cells, P-DRP1^S616^/P-DRP1^S637^ levels remained significantly higher in patient fibroblasts (Figure 8, D and E). In contrast, the protein levels of key fusion proteins MFN2, MFN1, and OPA1 remained unaltered in RINT1-mutated fibroblasts (Supplemental Figure 3, F and G). Together, these results indicate an imbalance of mitochondrial dynamics toward increased fission, which is likely the result of augmented levels of phosphorylated DRP1^S616^.

Our data indicate that RINT1 deficiency induces a secondary disruption of the mitochondrial dynamics and subsequent bioenergetic failure and oxidative stress. Mitochondrial impairment may also lead to the inadequate use of FAs as a carbon source for ATP production and the rerouting of FAs into LDs in a vicious cycle scenario. Moreover, our data indicate that glucose deprivation promotes LD accumulation and increases mitochondrial fragmentation, which could lead to greater toxicity in RINT1 cells.

## Discussion

We describe an early onset movement disorder presenting as complex HSP with ataxia, optic nerve atrophy, dysmorphic features, and developmental delay, thus broadening the previously described disease spectrum of ALF and skeletal abnormalities caused by RINT1 biallelic variants (29).

Of note, we show that RINT1 variants destabilize the NRZ-complex integrity by decreasing the protein levels of its subunits ZW10 and NBAS and disrupting the ER localization of ZW10. Notably, RINT1 mutations led to decreased protein levels of NBAS, which variants were identified initially as a cause of short stature, optic atrophy and Pelger Huet anomaly (SOPH syndrome, MIM no. 614800)(62), and later, of fever-triggered recurrent ALF (63–66). Since then, additional patients with NBAS deficiency displaying neurologic features including motor delay, muscular hypotonia, intellectual disability, and brain atrophy have been reported (63, 64, 67). Thus, our results suggest a convergent disease etiology for optic nerve atrophy, muscular hypotonia, neurodevelopmental delay, and liver failure phenotypes observed in RINT1- and NBAS-deficient patients.

In contrast, no significant differences were seen in NBAS levels in the initial study of the RINT1-linked ILFS3 (29), suggesting that the deficiency or instability of RINT1 in those patients was less deleterious for the NRZ complex. Here, the severity of the neurological symptoms appears to correlate well with the protein abundance of RINT1, NBAS, and ZW10, which suggests that loss of NRZ-complex integrity and function may be the origin of the neurological phenotype observed in our patients. Identification of additional patients and variants, as well as in-depth characterization of the precise impact of each novel mutation on NRZ stability will enable the definition of the phenotypic spectrum linked to *RINT1* variants, along with the establishment of genotype-phenotype correlations.

Importantly, the NRZ complex has been shown to play an essential role in ER-LD tethering and LD biogenesis, with depletion of *NBAS* or *ZW10* resulting in disturbed LD growth and lipid storage (36). In line with these findings, one of the most prominent features observed in this study is the accumulation of smaller lipid droplets in RINT1 fibroblasts. Growing evidence from human genetic studies has shown that key players at the LD-ER interface are a leading cause of neurological disease, more precisely of spastic paraplegias (9, 68). One of these is the NRZ-complex partner RAB18, causing Warburg Micro syndrome 3 (MIM no. 614222), a neurodevelopmental disorder of optic nerve atrophy, spastic quadriplegia, intellectual disability, microcephaly, hypoplasia of the corpus callosum, and hypogonadism (69). Thus, we posit that the RAB18-NRZ complex perturbations and subsequent abnormal ER-LD contact may contribute to the HSP phenotype in our patients.

Beyond the cellular phenotypes, the systemic lipid imbalance that RINT1 malfunction causes is striking. The failure of proper LD biogenesis may impact the synthesis of TGs, DGs, and phospholipids. Thus, the accumulation of phospholipids suggest that DGs may be shunted toward de novo phospholipid synthesis though the CDP-Ethanolamine Kennedy pathway due to an increase in *PCYT2* expression. Of note, the lysophospholipid levels and *PLA2G6* expression levels were drastically decreased in fibroblasts, indicating that RINT1 malfunction impacted the regulation of the Lands cycle. Indeed, loss of function of both *PCYT2* and *PLA2G6* leads to severe neurological disorders (7, 8, 70–72), with biallelic mutations in *PCYT2* causing a complex form of HSP with optic nerve atrophy, which is associated with neutral ether lipid and ether phospholipid abnormalities (17, 18, 73). PLA2G6 is essential for the remodelling of membrane phospholipids in the nervous system, which, in turn, can induce alterations in the regulation of mitochondrial physiology and the generation of ROS (74). Thus, the markedly reduced *PLA2G6* expression in RINT1 cells may contribute to mitochondrial malfunction.

We, therefore, posit that the mitochondrial fragmentation observed in RINT1-deficient fibroblasts may be ascribed to phospholipid composition changes at mitochondrial membranes. Phospholipids play key roles in mitochondrial dynamics by interacting with DRP1 and by directly changing the biophysical properties of mitochondrial membranes (75, 76). In particular, several studies have shown that PE regulates membrane fission and fusion events by increasing negative membrane curvature (77–79) and perturbs stability of mitochondria respiratory chain supercomplexes (80). In agreement with this, we found increased mitochondrial fission, defective cristae morphology, reduced mitochondrial membrane potential, and reduced ATP production in patient fibroblasts.

Phospholipid equilibrium is also essential for efficient autophagy. A recent report showed that lower PC/PS ratios have a detrimental effect on phagophore sealing and autophagosome maturation (81). In line with this study, our lipidomic analysis revealed a 60% drop in PC/PE ratio, correlating with inhibition of the autophagy flux in the cells of patients with a RINT1 mutation.

Altered lipid metabolism may also contribute to the liver failure seen in patients with *RINT1*-associated diseases. The hallmark of nonalcoholic fatty liver disease (NAFLD) is excessive hepatic accumulation of neutral lipids that results from altered lipid availability, including FA uptake and de novo lipogenesis, impaired utilization of FAs, including mitochondrial fatty acidβ-oxidation, or reduced lipid removal, including export as a component of VLDL particles (82). Our study revealed profound alterations in lipid metabolic pathways that could contribute to the pathogenesis of ALF: (a) a decrease in the PC/PE ratio, which compromises cell membrane integrity and results in hepatocyte damage and inflammation (83–85); (b) the inhibition of TG and DG synthesis and the increase in FC levels and FC/CE ratios in patient plasma, which may suggest alteration of hepatic cholesterol turnover, the key player of NAFLD (86); and, (c) the induction of the de novo lipogenesis pathway with activation of SREBP-1c, a major contributor to the development and severity of NAFLD (87).

Moreover, the increased DRP1-mediated mitochondrial fission may be critical for the progression of liver disease, since inhibition of mitochondrial fission prevented NAFLD by suppression of hepatic oxidative stress and lipid accumulation in primary hepatocytes (88). Lastly, lipophagy inhibition, as well as impairment of autophagic clearance of damaged mitochondria, could exacerbate the cellular buildup of abnormal organelles and contribute to a vicious cycle of cellular demise.

In conclusion, we describe what we believe to be a novel disease entity caused by RINT1 dysfunction — an early onset complex HSP — thereby expanding previous ALF and skeletal phenotypes. Our findings highlight the crucial role of RINT1 — and, more broadly, the NRZ complex — in neutral lipid and phospholipid metabolism and add a strong piece of evidence pointing at abnormal lipid homeostasis as a culprit in neurodevelopmental and movement disorders.

## Methods

### Clinical studies.

The patient identified as proband P1 was evaluated at University Hospital Germans Trias i Pujol (Badalona, Spain), whereas patient P3 visited the Neurology Departments of University Hospital Niño Jesús (Madrid, Spain) and Hospital Teresa Herrera (A Coruña, Spain). Information on the patient identified as proband P2 was retrieved from clinical records. Blood samples and skin-derived fibroblast cell lines were obtained using standard methods.

### WES.

Genomic DNA was extracted from peripheral blood using standard methods. WES was performed on the patient DNA samples using a SeqCap EZ Human Exome Kit v3.0 (Roche) for DNA capture. Exome sequencing was performed using a HiSeq 2000 Platform (Illumina) at the Centro Nacional de Análisis Genómico (CNAG, Barcelona, Spain). Variants were filtered using the RD-Cat platform (https://rdcat.cnag.crg.eu/) and an in-house pipeline based on GATK best practice guidelines. They were prioritized using an in-house bioinformatics pipeline (ClinPrior: https://github.com/aschluter/ClinPrior) based on accurate patient characterization with Human Phenotype Ontology (HPO) terms and interaction networks at physical and functional levels (30). This tool allowed us to identify the RINT1 variants through the combination of a phenotype-driven propagation network approach and a variant deleteriousness score based on the minor allele frequency (MAF) gnomAD allele frequency < 0.01, the variant impact prediction tool VEP (Variant Effect Predictor[PMID: 27268795]), CADD in silico damage predictor scores (89), and variant intolerance scores generated by the ExAC and gnomAD consortia (30). Sanger sequencing was used in all cases to confirm the findings and for family segregation. The variants were categorized according to ACMG/AMP criteria for pathogenicity (90, 91).

### Cell culture and treatment.

Primary skin fibroblasts were collected from patients P1 and P3 and anonymized people in the healthy control group (*n* = 6). More information about the control group can be found in Supplemental Table 2. The fibroblasts were cultured in parallel in DMEM (Gibco) supplemented with 10% FBS and 100 μg/mL penicillin–streptomycin at 37°C in a 5% CO_2_ atmosphere. For glucose deprivation experiments, cells were incubated for 16 hours in DMEM without glucose (Biological Industries) supplemented with L-glutamine, 10% FBS, and 100 μg/mL penicillin–streptomycin at 37°C in a 5% CO_2_ atmosphere.

For autophagy flux assay, cells were incubated in a minimal medium deprived of serum in presence or absence of 400 nM of Bafilomycin A1 (Merck) for 4 hours and prepared for Western blot analysis.

For temperature experiments, fibroblasts were seeded and allowed to adhere at 37°C. The cells were shifted to incubators set to 37°C or 40°C and incubated at the respective temperature for 18 hours.

### RNA splicing analysis.

RNA was extracted from primary fibroblasts using an RNeasy Mini Kit (Qiagen), and cDNA was synthesized using a SuperScript IV Kit (Life Technologies) following the manufacturer’s instructions. cDNA was amplified by PCR using primers targeting exons 10–14 of the *RINT1* gene (forward: 5′-TGAAAGTTCCAGATTGTGCAGA-3′; reverse: 5′-GGCTGCTCCTCCTTCATTGA-3′), and Sanger sequencing was performed.

### Western blotting.

Cell extracts were prepared in RIPA buffer (50 mM NaCl, 1% Nonidet P40 [Merck], 0.5% sodium deoxycholate [Merck], 0.1% SDS, 50 mM Tris, pH 8.0) supplemented with the Roche-cOmplete protease-inhibitor mix (Merck) and Halt phosphatase inhibitor cocktail (Thermo Fisher Scientific). The protein concentrations of the homogenates were determined using the Pierce BCA Protein Assay Kit (Thermo Fisher Scientific). Electrophoresis was carried out using SDS–PAGE NuPAGE Novex Bis-Tris gels (Invitrogen). The proteins were transferred onto nitrocellulose membranes (Bio-Rad) with an iBlot 2 Gel Transfer Device (Invitrogen) and analyzed with the required antibodies, listed below. The proteins were detected with an enhanced chemiluminescence Western blot detection system (GE Healthcare Bio-Sciences AB) and visualized with a ChemidocTouch Imaging System (Bio-Rad). The quantification of immunoblots was performed by densitometry using ImageLab Software (U.S. National Institutes of Health, USA).

For Western blotting, the following antibodies were employed: anti-RINT1 (Merck; catalog HPA019875; 1:1,000), anti-ZW10 (Proteintech, catalog 24561-AP, 1:1,000), anti-NBAS (Abcam, catalog ab122370, 1:1,000), anti-RAB18 (Proteintech, catalog 11304-1-AP, 1:1,000), anti-p62 (Abcam, catalog ab56416, 1:1,000), anti-LC3B (Cell Signaling Technology, catalog 2775, 1:1,000), anti-VDAC1 (Abcam, catalog ab15895, 1:1,000), anti-Complex III (Molecular Probes, catalog A21362, 1:1,000), anti-Complex IV (Invitrogen, catalog A21348, 1:1,000), anti-α-tubulin (Abcam; catalog ab80779; 1:10,000), anti-DRP1 (Cell Signaling Technology; catalog 14647; 1:1,000), anti-P-DRP1^s616^ (Cell Signaling Technology, catalog 3455; 1:1,000), anti-P-DRP1^s637^ (Cell Signaling Technology, catalog 6319, 1:1,000), anti-Mitofusin 1 (Proteintech ptglab.com, catalog 13798-1-AP, 1:1,000), anti-Mitofusin 2 (Sigma-Aldrich, catalog M6444, 1:1,000), anti-OPA1 (BD Transduction Laboratories, catalog 612607, 1:1,000), and HRP-conjugated anti-mouse and anti-rabbit antibodies (DakoCytomation; catalogs P0447 and P0448; 1:5,000–10,000).

### Microscopy analysis.

For immunostaining, cells were permeabilized and blocked in blocking buffer (1% BSA [Merck], 0.2% powdered milk, 2% NCS [Merck], 0.1 M glycine [Merck], 0.1% Triton-X-100 [Thermo Fisher Scientific]) for 15 minutes at 25°C. The cells were incubated with primary antibodies at 4°C overnight, washed with PBS, and incubated with secondary antibodies for 1 hour at room temperature.

For immunofluorescence, the following antibodies were employed: anti-RINT1 (Merck; catalog HPA019875; 1:200), anti-ZW10 (Proteintech, catalog 24561-AP, 1:200), anti-RAB18 (Proteintech, catalog 11304-1-AP, 1:1,000), anti-Calnexin (Novus biologicals, catalog NB300-518, 1:200), anti-TOMM20 (Abcam; catalog Ab56783; 1:200), anti-mouse conjugated to Alexa 647 (Thermo Fisher Scientific; catalog A21236; 1:1,000), and anti-rabbit conjugated to Alexa 488 (Thermo Fisher Scientific; catalog A11034; 1:1,000).

For staining of mitochondria, MitoTracker Orange (Thermo Fisher Scientific, catalog M7510) was used according to the manufacturer’s instructions. Briefly, MitoTracker Orange was diluted in prewarmed medium and added to the culture. After 15 minutes of incubation at 37°C, the cells were washed 3 times with PBS and fixed using 4% formaldehyde for 25 minutes.

For LD staining, a working solution of oil red O was made for each staining procedure and consisted of 100 mg oil red O (Merck) in 20 mL 60% triethyl phosphate (Merck). Fifteen milliliters of working solution was added to 8 mL dd H_2_O and filtered twice to remove any residual oil red O crystals. The cells were washed twice, fixed with 10% formaldehyde in PBS for 30 minutes, and washed twice again. The cells were then stained with Oil Red O working solution for 15 minutes at room temperature in the dark and washed twice before microscopic analysis.

All images were acquired using a Leica TCS SL laser scanning confocal spectral microscope (Leica Microsystems Heidelberg GmbH) with a 63 × oil immersion objective lens.

For the fluorescence quantification of ER-RINT1 colocalization, the coloc2 function of ImageJ software was used to calculate Pearson’s correlation coefficient. The LD number and area and the mitochondrial number and average size were scored using the Analyse Particle function of ImageJ software.

For 3D reconstruction, confocal image stacks of cells stained with anti-TOMM20 antibody, for the mitochondria, and Oil Red O for the LDs, were imported to Imaris 9.7.2 (Bitplane), and the surfaces tool was used to create an isosurface for each channel. Unwanted isosurfaces were filtered out using the Imaris filter tool. The distances between surfaces were calculated by Imaris, and a surface-surface filter (“shortest distance to surfaces-surfaces”, value ≤ 0.0344 μm) was used to determine the number of LDs in contact with the mitochondria surface. The number and area of LDs interacting with mitochondria were collected and selected for statistical analyses. LDs with different areas (0–1, 1–2, and > 2 μm^2^) were quantified, and the values were averaged for each cell.

### Electron microscopy.

The fibroblasts were harvested, and the pellet was fixed in 2.5% glutaraldehyde (EMS) and postfixed with 1% osmium tetroxide (OsO4; EMS). Cells were dehydrated using a grades ethanol series and then embedded in the epoxy resin (Ted Pella), Ultrathin sections (80 nm) were cut by a Leica EM UC6 (Leica). Sections were contrasted with 6% uranyl acetate followed by 2% lead citrate (EMS) and examined in a Jeol JEM-1010 80kv transmission electron microscope (TEM). Digital images were taken by using a CCD Orius camera (GATAN).

### Lipidomics profiling.

Lipidomics experiments were performed at CSIC (Barcelona, Spain). Whole blood was obtained from controls and patients with *RINT1* mutations after an overnight fast and collected in EDTA tubes. The blood samples were centrifuged at 400*g* for 30 minutes using a gradient of Histopaque (Merck) to separate plasma, erythrocytes, and peripheral blood mononuclear cells (PBMCs). Confluent fibroblasts were harvested using trypsin and pelleted at 1500*g* for 5 minutes to obtain a pellet containing one-million cells.

Lipids were analyzed as described previously (92) with minor modifications. In detail, for phospholipids and neutral lipids, a total of 750 μl of a methanol-chloroform (1:2, vol/vol) solution containing internal standards (16:0 D31_18:1 phosphocholine, 16:0 D31_18:1 phosphoethanolamine, 16:0 D31_18:1 phosphoserine, 17:0 lysophosphocholine, 17:1 lysophosphoethanolamine, 17:1 lysophosphoserine, 17:0 D5_17:0 diacylglycerol, 17:0/17:0/17:0 triacylglycerol and C17:0 cholesteryl ester; 0.2 nmol each from Avanti Polar Lipids) was added to 0.002 mL of plasma or lysate from one-million fibroblasts. The samples were vortexed and sonicated until they appeared dispersed and were extracted at 48°C overnight. The samples were then evaporated and transferred to 1.5 mL eppendorf tubes after the addition of 0.5 mL of methanol. The samples were evaporated and stored at –80°C until analysis. Before analysis, 150 μl of methanol was added to the samples, and the samples were centrifuged at 13,000*g* for 3 minutes. Then, 130 μl of the supernatant was transferred to ultra-performance liquid chromatography (UPLC) vials for injection and analysis.

All lipids were analyzed by liquid chromatography-high resolution mass spectrometry (LC-HRMS) using an Acquity ultra high-performance liquid chromatography (UHPLC) system (Waters) connected to a time-of-flight detector (LCT Premier XE). Full-scan spectra from 50 to 1800 Da were acquired, and individual spectra were summed to produce data points of 0.2 seconds each. Mass accuracy at a resolving power of 10,000 and reproducibility were maintained by using an independent reference spray via LockSpray interference. Lipid extracts were injected onto an Acquity UHPLC BEH C8 column (1.7 μm particle size, 100 mm × 2.1 mm, Waters, Ireland) at a flow rate of 0.3 mL/minute and a column temperature of 30°C. The mobile phases were methanol with 2 mM ammonium formate and 0.2% formic acid (A)/water with 2 mM ammonium formate and 0.2% formic acid (B).

Positive identification of compounds was based on accurate mass measurements with an error of B5 ppm and an LC retention time of 92% compared with that of a standard. Quantification was carried out using the extracted ion chromatogram of each compound using 50 mDa windows. The linear dynamic range was determined by injecting mixtures of internal and natural standards as indicated above. Since standards for all identified lipids were not available, the amounts of lipids are given as pmol equivalents relative to each specific standard or as pmol/mg protein.

### RNA extraction and real-time PCR.

Total RNA was extracted using an RNeasy Kit (Qiagen) according to the manufacturer’s instructions. RNA was transcribed into complementary DNA (cDNA) using SuperScript II reverse transcriptase (Invitrogen). Real-time PCR (RT-PCR) was performed in a LightCycler 480 RT-PCR System (Roche Diagnostics GmbH). Primers for human *PCYT2* (F-5′-CTCACCACAGACCTCATCGT-3′, R-5′-TGCCAGGTTAGAAGTCACCA-3′), *SREBP-1c* (F-5′-GGAGGGGTAGGGCCAACGGCCT-3′, R-5′-CATGTCTTCGAAAGTGCAATCC-3′), and *FASn* (F-5′-CGCGTGGCCGGCTACTCCTAC-3’, R-5′-CGGCTGCCACACGCTCCTCT-3′) were designed (Invitrogen). Standardized primers for human *DGAT1* (HS00201385_M1), *DGAT2* (HS01045913_M1), *PLA_2_G6* (Hs00895669_m1) and *RPLP0* (HS99999902_M1) were used (Custom TaqMan Gene Expression Assays; Applied Biosystems).

Each sample was run in triplicate, and the mean value was used to calculate the mRNA expression using the comparative (2−ΔCt) method according to the manufacturer’s instructions.

### Adenosine triphosphate measurement.

ATP levels were measured by chemiluminescence using an ATPlite 1step system (PerkinElmer) according to the manufacturer’s protocol and normalized to the total protein concentration, as previously described (93).

### Inner mitochondrial membrane potential quantification by flow cytometry.

Confluent cells were washed with PBS and incubated with 50 nM TMRE (Molecular Probes) in prewarmed PBS for 30 minutes at 37 °C. Flow cytometry analysis was performed on a Gallios analyser (Beckman Coulter) equipped with a blue 488 nm solid-state laser as an excitation source, as described previously (94). Fluorescence was measured in the FL3 channel using 620/30 bandpass filters for the TMRE probe. A total of 20,000 cells were analyzed for each sample. Histograms showing the percentage of depolarized cells were obtained after gating live cells. The data were analyzed with FlowJo Tree Star software.

### Intracellular ROS measurement.

Intracellular ROS levels were estimated using the ROS-sensitive H_2-_DCFDA probe (Thermo Fisher Scientific) as previously described (95). Following incubation with 10 μM H_2-_DCFDA for 30 minutes, the cells were washed twice with PBS and lysed with 1% Triton. The fluorescence of H_2-_DCFDA-stained cells was measured with a spectrofluorometer (excitation wavelength 493 nm, emission wavelength 527 nm). The ROS levels were normalized to the total protein concentration.

### Data availability.

The *RINT1* variants have been reported to the NCBI’s public ClinVar archive (https://www.ncbi.nlm.nih.gov/clinvar/) under accession numbers SCV003918812, SCV003918811, SCV003918810.

### Statistics.

Data are presented as the mean ± SD and *P* values less than 0.05 were considered significant. All statistical analyses were performed using GraphPad Prism (GraphPad Software). Statistical significance was assessed by 2-tailed Student’s *t* test and 1- or 2-way ANOVA with Tukey’s multiple-comparison test.

### Study approval.

This study was conducted in accordance with the Declaration of Helsinki and approved by the Clinical Research Ethics Committee of Bellvitge (PR076/14). The patients and their relatives provided written informed consent for the collection, storage, and publication of the clinical data, blood samples, and experimental results under the approval of the Clinical Research Ethics Committee of Bellvitge (PR076/14). All clinical photographs appear with parental informed written consent.

## Author contributions

NL and AP conceptualized the project. AP acquired funding for the project. NL, LPS, CG, LG, MR, JC, EV, AS, and EJ conducted research and performed the experiments. ARP, MVC, LGGS, AP, OBT, and CC were responsible for the clinical studies. EAG and AP supervised the project. NL, MR, EAG, and AP wrote the original draft of the manuscript. FC and JLC helped with data interpretation, edited the manuscript, and brought forth important intellectual input.

## Supplementary Material

Supplemental data

## Figures and Tables

**Table 1:**
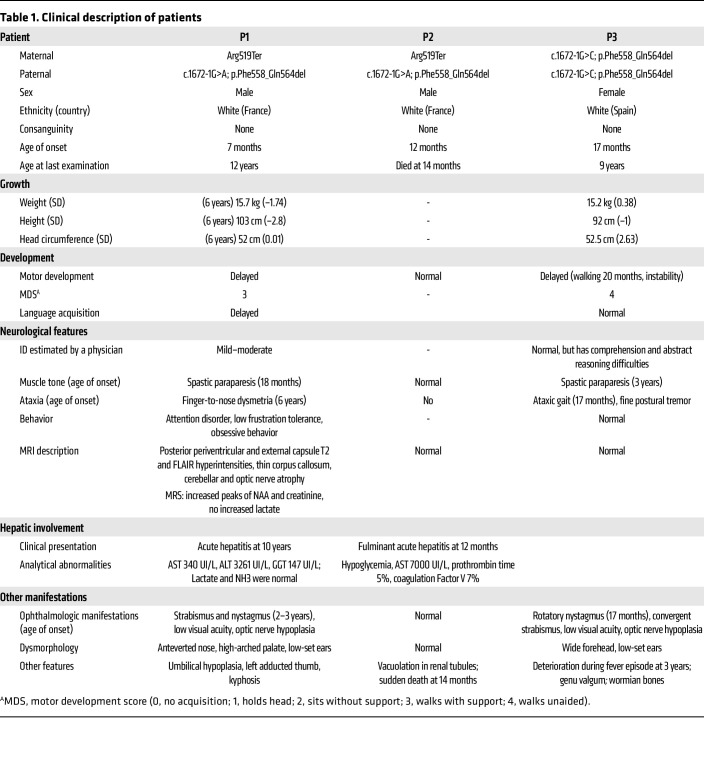
Clinical description of patients

## Version 1 07/17/2023

Electronic publication
