# Supplementary material for: RINT1 deficiency disrupts lipid metabolism and underlies a complex hereditary spastic paraplegia
Source: J Clin Invest. 2023 Jul 17;133(14):e162836. doi: 10.1172/JCI162836 (PMC10348772; doi:10.1172/JCI162836)
Supplement: Supplemental data [file jci-133-162836-s093.pdf]

SUPPLEMENTAL MATERIAL

Clinical features of RINT1 patients

Family A: Affected siblings P1 and P2 born to French parents.

Patient P1: the pregnancy course featured maternal hypertension from the fourth month and intrauterine growth restriction. At 38 weeks of gestation, an emergent cesarean section was performed due to abnormal cardiotocography tracing. The patient had a birth weight of 2440 g (-1.75 SD), short stature (45 cm; -2.6 SD) and his head circumference was 32 cm (-1.26 SD). He presented early motor developmental delay with poor head control at 3 months, acquisition of stable sitting at 10 months, and he never walked independently. Physical examination revealed an anteverted nose, high-arched palate, low-set ears, left adducted thumb, umbilical hypoplasia, and kyphosis (Figure 1B). Axial hypotonia and progressive spastic paraparesis was evident at 18 months. At three years of age, the ophthalmologic exam revealed convergent strabismus with nystagmus, optic nerve pallor, and visual acuity 3/10. The optic nerve atrophy worsened at five years of age, with clearly abnormal visual evoked potentials and normal electroretinogram. At 6 years of age, he showed hand motor difficulties, finger-to-nose dysmetria and scissor gait with brisk deep tendon reflexes, bilateral ankle clonus and Babinski sign. He also had a worsening of sphincter control at that age. He received botulinum toxin treatment between the ages of 3 and 8 years, and was able to walk with the aid of a walker, while he used a wheelchair outdoors. Since he was 10 years old, he uses a wheelchair permanently, despite the implantation of a baclofen pump at 12 years. He also exhibited mild-moderate intellectual disability, with a severe attentional and executive disorder but a good level of language, and behavioral traits such as stubbornness, obsessiveness, and low frustration tolerance.

An MRI study performed at 2 years of age revealed posterior periventricular and external capsule hyperintensities in T2 and FLAIR sequences, probably corresponding to delayed myelination, and thinning of the corpus callosum. The spinal MRI was normal. At 5 years of age, MRI showed an enhancement of posterior periventricular hyperintensity and signs of cerebellar, and optic nerves and chiasm atrophy (Figure 1C). Spectroscopy revealed increased peaks of N-acetylaspartate (NAA) and creatinine, without increased lactate. Spinal radiographs showed large lumbar vertebrae. Metabolic studies showed a mild decrease in the plasma free carnitine level, whereas plasma lactate and CSF glucose, lactate and pyruvate levels were all normal. Brainstem auditory evoked potentials, EEG, and peripheral nerve conduction studies were normal.

At 10 years of age, he was hospitalized due to acute hepatitis with vomiting and fever. Complementary exams performed at that moment showed AST 340 UI/L, ALT 3261 UI/L, and GGT 147 UI/L. Lactate and NH_3 were normal.

Patient P2: his gestation and birth were uneventful. He showed short stature at birth (46 cm; -2.4 SD). However, his birth weight and head circumference were normal. He showed normal neurological development until he suffered fulminant hepatitis and hepatic encephalopathy (HE) in the context of an enterovirus infection when he was 1 year old. The episode was associated with hypoglycemia, AST 7000 UI/L, prothrombin time 5%, and coagulation factor V 7%, but total bilirubin was normal. Metabolic studies showed increased ammonia (600 μmol/L) and lactate (5 mmol/L) and low carnitine levels. However, acylcarnitines, free fatty acids, urine succinylacetone and autoimmunity markers (antinuclear antibodies (ANA), smooth muscle antibodies (SMA), antibodies to liver kidney microsome type-1 (LKM1), antibodies to soluble liver antigen (SLA)/liver pancreatic antibodies (LP), antibodies to liver cytosol antigen 1 (LC1) and anti-

mitochondrial antibodies (AMA)) were normal. The appearance of this Reye-like episode with hyperlactatemia raised the suspicion of a mitochondrial disorder. Cranial MRI, cardiac ultrasound and skeletal radiographs were normal. After recovery from that episode with normalization of transaminases, he suffered sudden death at 14 months of age. Postmortem microscopic examination revealed the presence of hepatic steatosis and vacuolization of the proximal tubules of both kidneys.

Family B: patient P3 is a female born to Spanish parents. Her gestation, birth, and family history were unremarkable. Although this patient did not show any neurodevelopmental abnormalities, she had unsteady gait with frequent falls at the age of 17 months. Nystagmus was evidenced in association with stress or fever, and fixation and extreme gaze. At 3 years old, she suffered a general deterioration for three days in the context of a febrile illness. Physical examination revealed a wide forehead, low-set ears, convergent strabismus of the left eye, a spastic-ataxic gait with brisk tendon reflexes, ankle clonus and Babinski sign (Figure 1B). Fundoscopic examination showed optic nerve hypoplasia. A tomography scan of the head showed numerous Wormian bones. She displayed normal cognitive capacities, except for comprehensive and abstract reasoning difficulties that required school reinforcement. The results of plasma, urine and cerebrospinal fluid metabolic tests, cranial and spinal MRI, and neurophysiological studies, including evoked potential and peripheral nerve conduction studies, were all normal.

SUPPLEMENTAL TABLES

Supplemental Table 1: features of *RINT1* variants identified in patients.

Variants	1	2	3
**Genomic position GRCh37**	Chr7:105195558C>T	Chr7:105204179G>A	Chr7:105204179G>C
**cDNA change NM_021930.6**	c.1555C>T	c.1672-1G>A	c.1672-1G>C
**Protein change NP_068749.3**	p.(Arg519Ter)	p.(Phe558_Gln564del)	p.(Phe558_Gln564del)
**Frequency in gnomAD (v2.1.1) (#total alleles)**	1.41E-05<br>(4 heterozygous in 282788 alleles)	ND	7.2E-06<br>(2 heterozygous in 277684 alleles)
**CADD_PHRED (score) (GRCh37-v1.6)**	41	35	35
**PredictSNP2 (score) (% expected accuracy)**	1<br>81%	1<br>89%	1<br>89%

Supplemental Table 2: control fibroblast features.

ID	Gender	Age (years)	Race	Passage number
**CTL1**	Male	16	Caucasian	13-16
**CTL2**	Male	17	Caucasian	13.16
**CTL3**	Male	18	Caucasian	14-17
**CTL4**	Male	26	Caucasian	13-16
**CTL5**	Male	22	Caucasian	13-16
**CTL6**	Male	25	Caucasian	11-13

SUPPLEMENTAL FIGURES

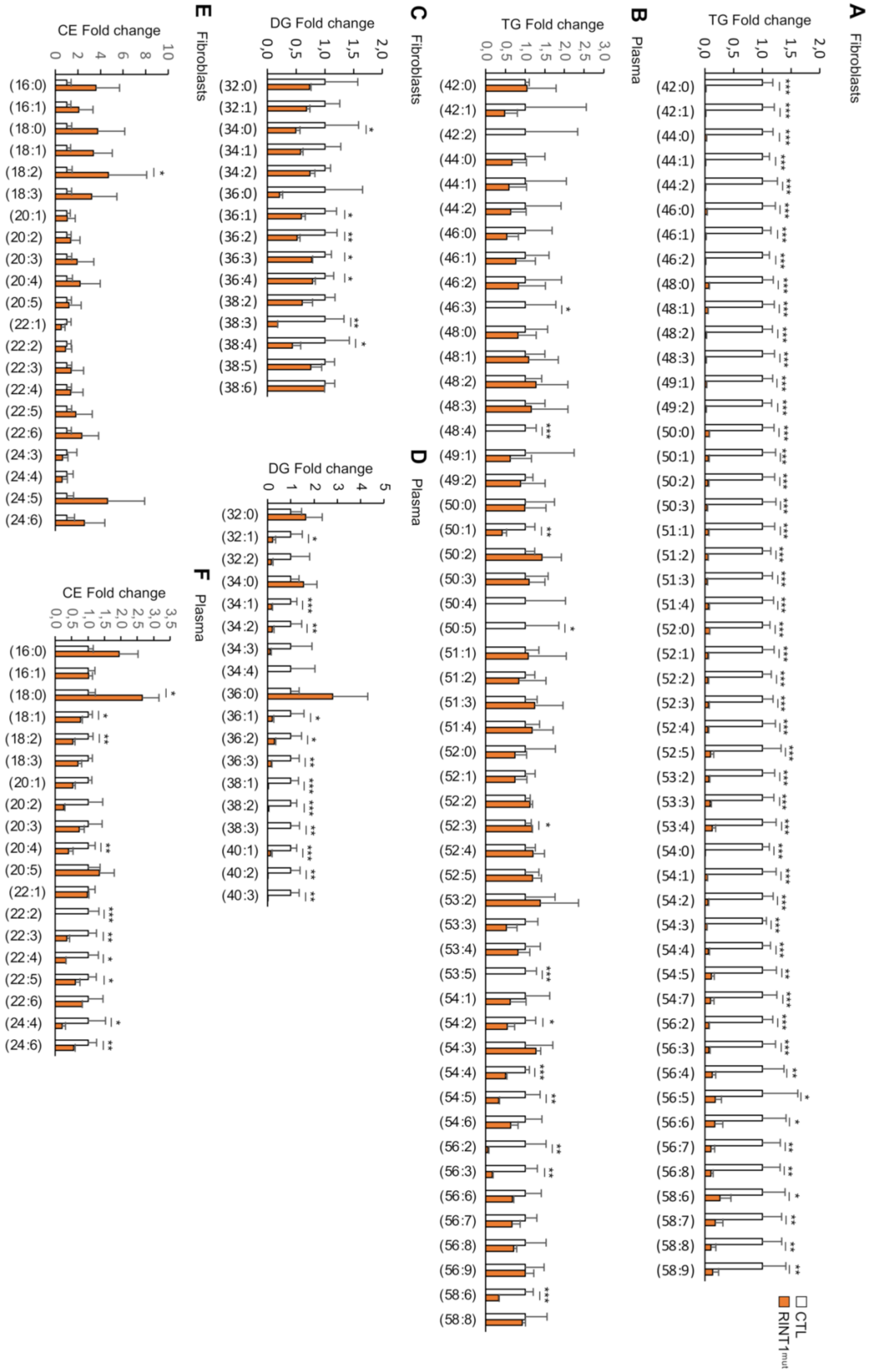

Supplemental Figure 1: the lipid molecular species detected in each class of neutral lipids in fibroblasts and plasma from patients P1 and P3 (RINT1^{mut}, n=2) relative to control (CTL, n=5-6) individuals ((**A-B**) triglyceride (TG); (**C-D**) diglyceride (DG); and (**E-F**) cholesterol ester (CE)). All data are shown as the mean ± SD. $*P < 0.05$, $**P < 0.01$, $***P < 0.001$. Data in were analyzed using unpaired 2-tailed *t test*.

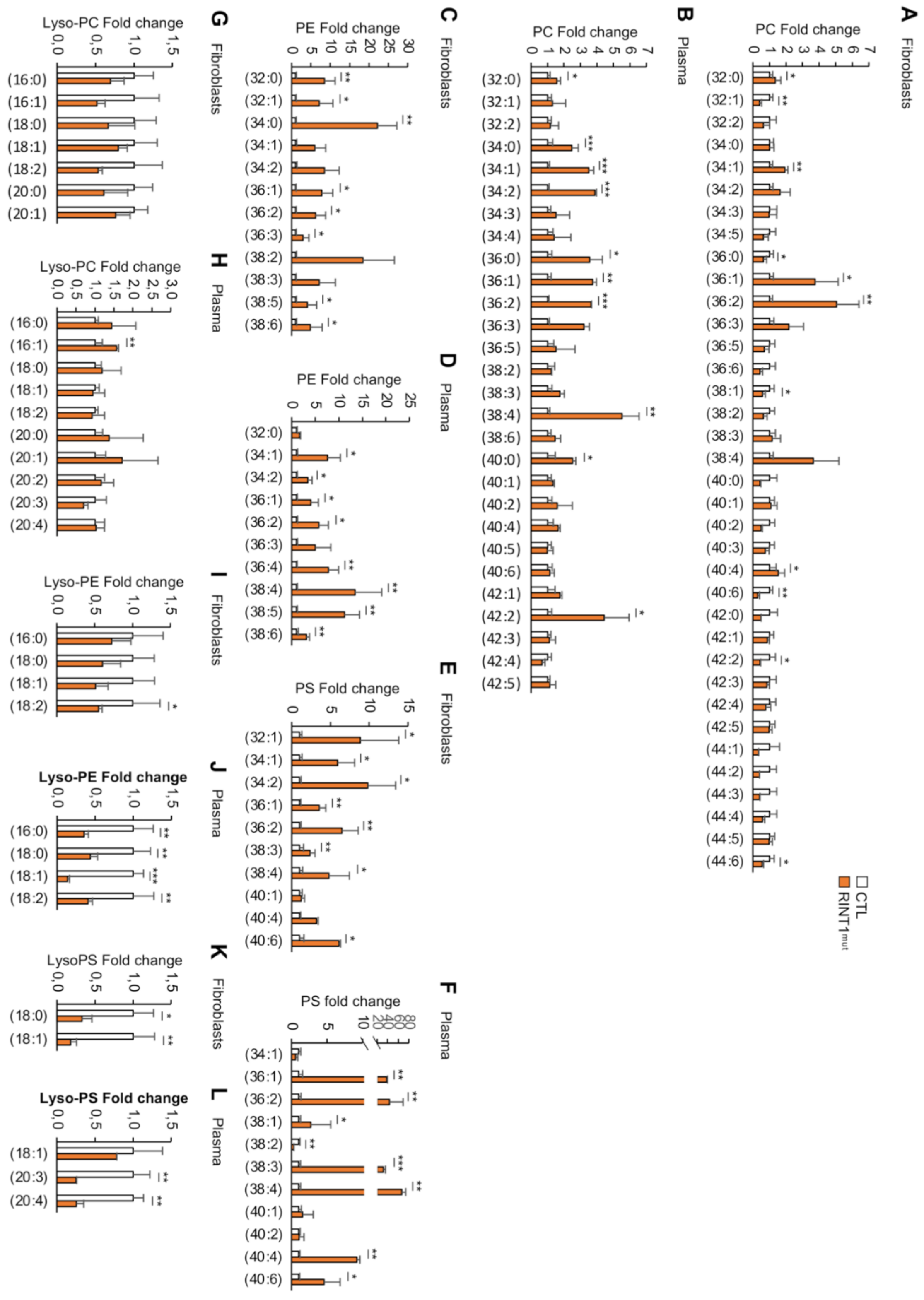

Supplemental Figure 2: the lipid molecular species detected in each class of phospholipids and lysophospholipids in fibroblasts and plasma from patients P1 and P3 (RINT1^{mut}, n=2) relative to control (CTL, n=5-6) individuals ((**A-B**) phosphatidylcholine

(PC); (**C-D**) phosphatidylethanolamine (PE); (**E-F**) phosphatidylserine (PS); (**G-H**) lysophosphatidylcholine (Lyso-PC); (**I-J**) lysophosphatidylethanolamine (Lyso-PE); and (**K-L**) lysophosphatidylserine (Lyso-PS). All data are shown as the mean ± SD. *P < 0.05, **P < 0.01, ***P < 0.001. Data were analyzed using unpaired 2-tailed *t test*.

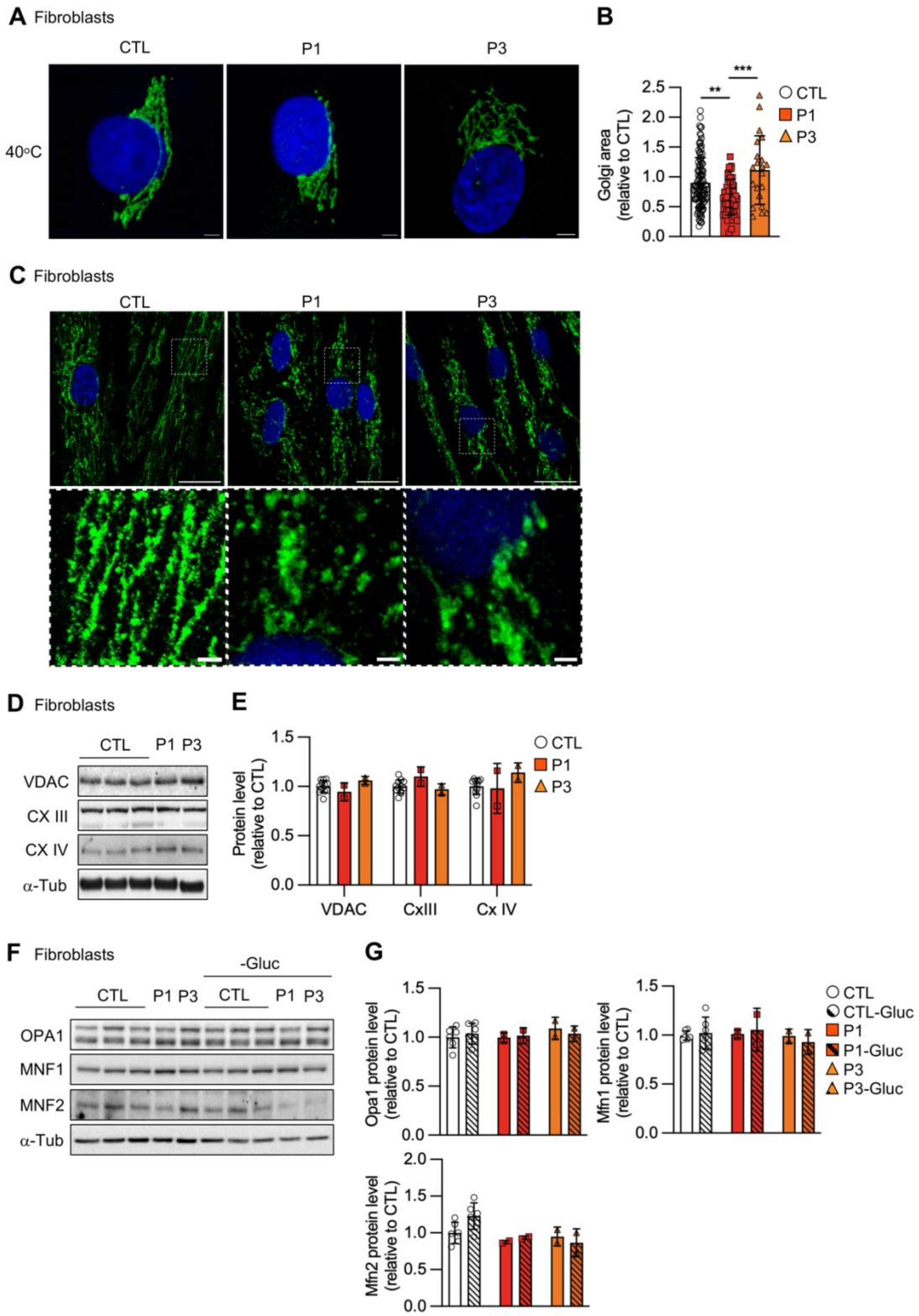

Supplemental Figure 3. (**A**) Representative images of Golgi apparatus of control (CTL) and patient (P1 and P3) fibroblasts incubated at 40ºC. Scale bars: 5 µm. (**B**) Quantification of Golgi area relative in patient fibroblasts (P1 and P3) to control cells (CTL, n=3). n>50

cells for each genotype. (**C**) Representative images of mitochondria labeled with an anti-TOMM20 antibody from control (CTL) and patient (P1 and P3) fibroblasts. Scale bar=20 μm. A zoomed-in view is shown for each image with a scale bar of 2 μm. (**D**) Control (CTL) and patient (P1 and P3) fibroblasts were subjected to immunoblot analysis using the anti-VDAC, anti-CxIII and anti-CxIV antibodies. The total amount of tubulin (α-Tub) was used as a loading control. Blots run in parallel using identical samples are shown. (**E)** Quantification of VDAC, CxIII and CxIV protein levels in patient cells (P1 and P3) relative to the controls (CTL, n=6). (**F**) Control (CTL) and patient (P1 and P3) fibroblasts were subjected to immunoblot analysis using the anti-OPA1, anti-Mfn1 and anti-Mfn2 antibodies. The total amount of tubulin (α-Tub) was used as a loading control. Blots run in parallel using identical samples are shown. (**G**) Quantification of OPA1, Mfn1 and Mfn2 protein levels in patient fibroblasts (P1 and P3) relative to the controls (CTL, n=6). All data are shown as the mean ± SD. Results were obtained from 2 independent experiments. $*P < 0.05$, $**P < 0.01$, $***P < 0.001$. Analysis of data in **B** and **E** were performed using 1-way ANOVA followed by Tukey's test for multiple comparisons. The data in **G** were analyzed by 2-way ANOVA followed by Tukey's test for multiple comparisons.
